# Impact of Tilapia hepcidin 2-3 dietary supplementation on the gut microbiota profile and immunomodulation in the grouper (*Epinephelus lanceolatus*)

**DOI:** 10.1038/s41598-019-55509-9

**Published:** 2019-12-13

**Authors:** Chen-Hung Ting, Chieh-Yu Pan, Yi-Chun Chen, Yu-Chun Lin, Tzong-Yueh Chen, Venugopal Rajanbabu, Jyh-Yih Chen

**Affiliations:** 1 Marine Research Station, Institute of Cellular and Organismic Biology, Academia Sinica, 23-10 Dahuen Road, Jiaushi, Ilan 262 Taiwan; 2 Department and Graduate Institute of Aquaculture, National Kaohsiung University of Science and Technology, Kaohsiung, 811 Taiwan; 3 Institute of Biotechnology & Department of Biotechnology and Bioindustry Sciences, National Cheng Kung University, Tainan, 701 Taiwan; 4 Anbil Dharmalingam Agricultural College and Research Institute, Tamil Nadu Agricultural university, Tiruchchirapalli, 620027 Tamil Nadu India; 5 The iEGG and Animal Biotechnology Center, National Chung Hsing University, Taichung, 402 Taiwan; 6 Center of Excellence for the Oceans, National Taiwan Ocean University, Keelung, 202 Taiwan

**Keywords:** Infection, Antimicrobial resistance

## Abstract

Hepcidin regulates iron homeostasis and host-defense mechanisms, while the hepcidin-like protein, Tilapia hepcidin (TH)2-3, functions as an antimicrobial peptide (AMP). Since AMP dietary supplements may be used as alternatives to antibiotics in livestock, we tested the effects of recombinant (r)TH2-3 as a dietary supplement in grouper aquaculture. rTH2-3 was produced by a *Pichia pastoris* expression system and exhibited thermostability and broad-spectrum antimicrobial activity. The feed conversion ratio and feed efficiency were determined in *Epinephelus lanceolatus* (grouper) fed with rTH2-3-supplemented diet for 28 days. In addition, grouper showed enhanced superoxide dismutase (SOD) activity after rTH2-3 feeding compared to regular-diet-fed fish. Gut microbiota analysis revealed that microbial diversity was enhanced by feeding grouper with 1% rTH2-3. After challenging grouper with *Vibrio alginolyticus*, differential regulation of immune-related genes in the liver and spleen was observed between the TH2-3 and regular-diet groups, including for genes associated with antimicrobial and pro-inflammatory functions, complement components, and major histocompatibility complex (Mhc). These findings suggest that overall immunity was improved. Thus, our results suggest long-term supplementation with rTH2-3 may be beneficial for aquacultured grouper. The beneficial effects of the supplement are likely based on changes in the commensal microbial community as well as immunomodulation.

## Introduction

Antibiotic abuse may lead to multidrug-resistant bacteria and antimicrobial-resistant infections, making inappropriate use of antibiotics an important issue in industrial farming, such as aquaculture^1^. One solution to mitigate the effects of antibiotic abuse is to identify antibiotic alternatives. Antimicrobial peptides (AMP) function in innate immunity and are conserved across species^2^. Most AMPs are structurally characterized as amphiphilic molecules with precise spatial arrangement of hydrophobic and hydrophilic residues^3–5^. Furthermore, these molecules show broad-spectrum antibacterial activity toward both Gram-positive and Gram-negative bacteria^4–8^, including antibiotic-resistant strains^9–11^. Hepcidin is an iron-regulatory hepatic peptide hormone that controls iron homeostasis (*i.e*. absorption, recycling and storage) in mammals^12,13^ and drives changes in the balance between commensal microbes and immunity^14,15^. Additionally, the *Hepcidin* gene is upregulated by bacteria or virus infection in the seabream, suggesting a role in the host-protective response^16^. Interestingly, synthetic seabream Hepcidin exerts *in vitro* antimicrobial activity against bacterial strains isolated from Seabream skin (Pdp11 and 51M6, members of the *Vibrionaceae* family in the genus *Shewanella*), *Bacillus subtilis*, and *Escherichia coli*^16^. Our group identified three *Hepcidin-*like genes (*TH1–5*, *TH2–2*, and *TH2-3*) in Nile tilapia (*Oreochromis mossambicus*), among which mRNA expression of *TH2-3* was elevated after lipopolysaccharides (LPS) challenge. Synthesized TH2-3 peptide further showed antimicrobial activity against *Vibrio damsela* and *Vibrio vulnificus*^3^. Moreover, *TH2-3*-transgenic zebrafish are resistant to *V. vulnificus* (204) challenge, suggesting that *TH2-3* expression can effectively inhibit bacterial growth *in vivo*^7^. Finally, the TH2-3 peptide was shown to protect mice from *V. vulnificus* infection and modulate expression of immune-related genes in bacteria-infected mice^6^.

Dietary supplementation with various AMPs has been reported to enhance host bacterial resistance, growth performance, and the gut microbe community. For example, feed supplemented with AMP-A3 enhanced growth performance and nutrient retention, while improving intestinal morphology in broilers^17^ and weanling pigs^18^. Supplementation with the *E. coli*-derived AMP, Microcin J25 (MccJ25), effectively improved performance, attenuated inflammation, and improved fecal microbiota composition of weaned pigs^19^. In another example, a 1:1 mixture of recombinant AMPs derived from swine and fly was used as a feed additive, conferring improved growth performance and enhancing rumen microorganism diversity in juvenile goats^20^. We have also reported that a recombinant version of fish-derived Epinecidin-1 may be used as a feed additive to improve bacterial resistance and enhance immune-related gene expression^8,21,22^. Together, these studies show that AMP-based dietary supplementation can have beneficial effects in a wide variety of animals. Furthermore, the biosafety of exogenous *Pseudosciaena crocea* hepcidin has been demonstrated in rats, where the gut microbiota population was also influenced by daily oral administration^23^. However, Hepcidin has not been previously investigated for its utility as a feed supplement in aquaculture.

The widely distributed grouper, *Epinephelus lanceolatus*, is a vulnerable species of high economic value. It was listed as a threatened species by the International Union for Conservation of Nature and Natural Resources^24^ due to loss of habitat and overfishing^25^. Successful aquaculture of this species has been accomplished in Taiwan, Indonesia, and China in the past decades^24^. A serious challenge in aquaculture that may cause huge economic loss is vibrosis^26^. *V. alginolyticus* is one of the major pathogens that causes vibriosis in *Epinephelus* sp.^27^. Antibiotic treatment in aquaculture to resolve ongoing infections is usually ineffective and has caused the enrichment of multiple antibiotic‐resistant strains of *Vibrio* sp.^27,28^. Medicated feed (*i.e*. mixing feed with antibiotics) is usually applied in aquaculture to maintain good health under environmental stresses, including high fish density, inadequate or poor nutrition, and microbial infection; however, this practice still promotes the growth of antibiotic-resistant strains, raising major public health concerns^29^. Therefore, treatments are mostly effective when administered prior to or at the very early stages of infection. In this study, we asked whether administration of TH2-3 to grouper may contribute to establishing a protective immune status, reflected by the commensal microbial community in the host. We first produced recombinant TH2-3 (rTH2-3) using a *Pichia Pastoris* system. rTH2-3 was then used as a feed additive for the culture of grouper. We investigated the growth performance, bacterial gut microbiota, and immune-related gene profiles upon *V. alginolyticus* challenge.

## Results

### Expression of recombinant rTH2-3

**Figure 1:**
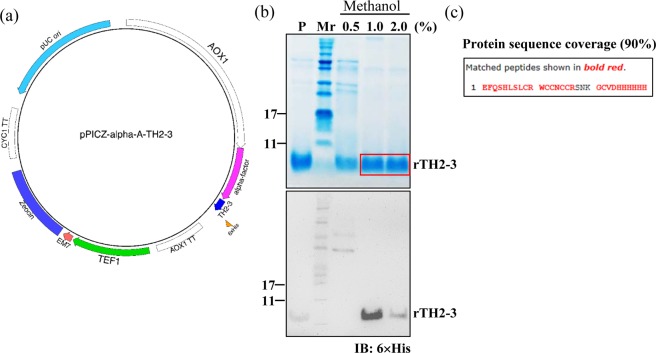
Expression and purification of rTH2-3. (**a**) The PICZ-α-A yeast expression system was used in this study. Codon-optimized TH2-3 sequence with a C-terminal 6×His tag was expressed. (**b**) Upper panel: SDS-PAGE analysis of supernatant collected from yeast culture with appropriate percentage of methanol. Boxed region was excised for in-gel digestion followed by LC-MS/MS analysis. Lower panel: Western blot analysis using antibody against the 6×His tag. (**c**) Protein database identification confirmed the excised protein band as TH2-3. “P” and “Mr” shown in (c) indicate positive control (synthesized TH2-3 peptide) and the molecular weight marker, respectively.

**Figure 2:**
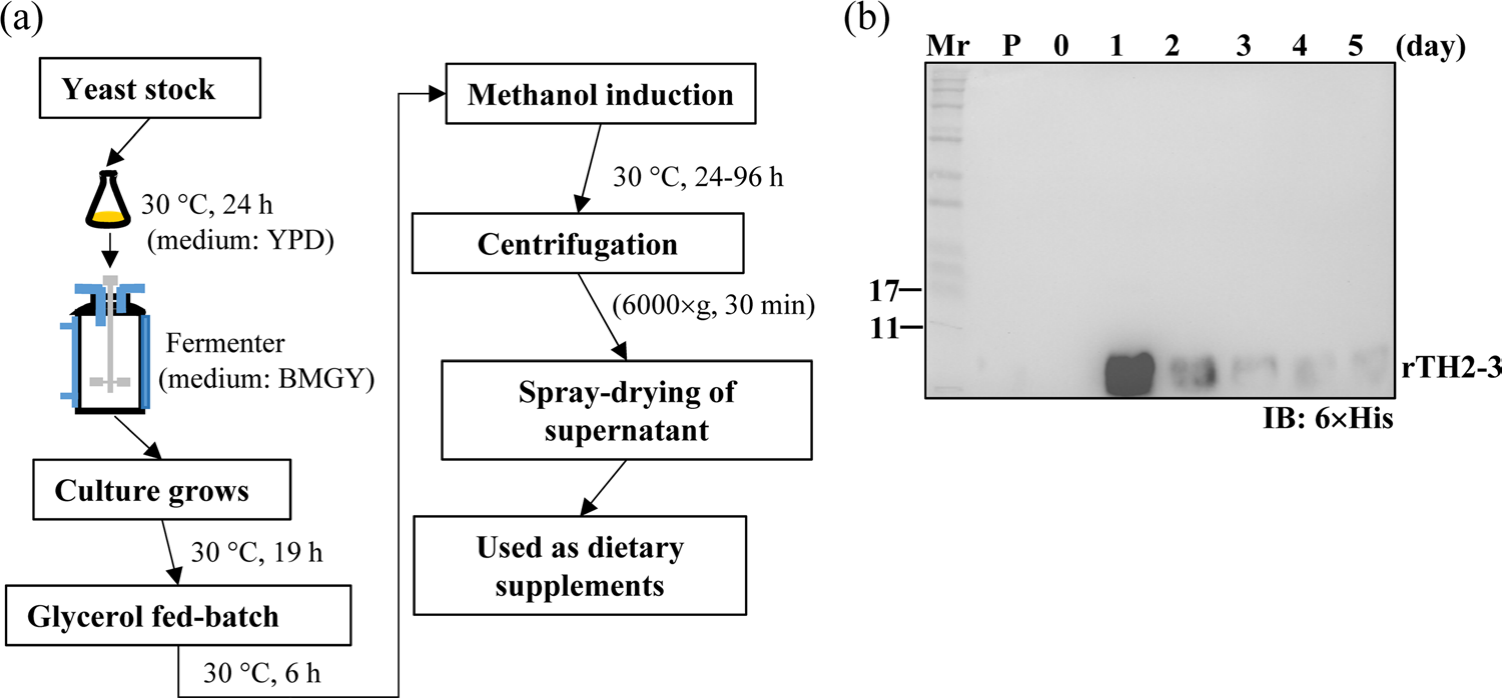
Production of rTH2-3 in fermenter cultures. (**a**) The procedures and conditions of small scale production of rTH2-3 using fermenter. (**b**) Western blot analysis confirmed that a large amount of rTH2-3 is produced at 24 h post-methanol addition (Day 1). “P” and “Mr” shown in (**b**) indicate positive control (synthesized TH2-3 peptide) and the molecular weight marker, respectively.

The *Pichia* vector was used to express a C-terminal 6×His-tagged, sequence-optimized TH2-3 that was generated by a full-gene synthesis approach (Fig. 1a). To optimize the methanol-induction conditions, culture medium was supplemented to 0.5–2.0% methanol, and protein induction was analyzed by SDS-PAGE and Western blot (Fig. 1b,c). The results show that 1% methanol was sufficient to maximally induce rTH2-3, as evidenced by a strong signal from the 6×His tag antibody (Fig. 1b, bottom). The gel band was excised from the SDS-PAGE for LC-MS/MS analysis (Fig. 1b, upper) and confirmed to be TH2-3 (Fig. 1c). For large-scale production of rTH2-3, an overnight culture of a small volume of yeast was moved to a fermenter for amplification (Fig. 2a). To test the best conditions for rTH2-3 production, culture supernatant was collected from day 1 to 5 of methanol induction for Western blot analysis. Methanol induction for 1 day produced the maximum amount of rTH2-3 (Fig. 2b).

### Antimicrobial activity of rTH2-3

**Figure 3:**
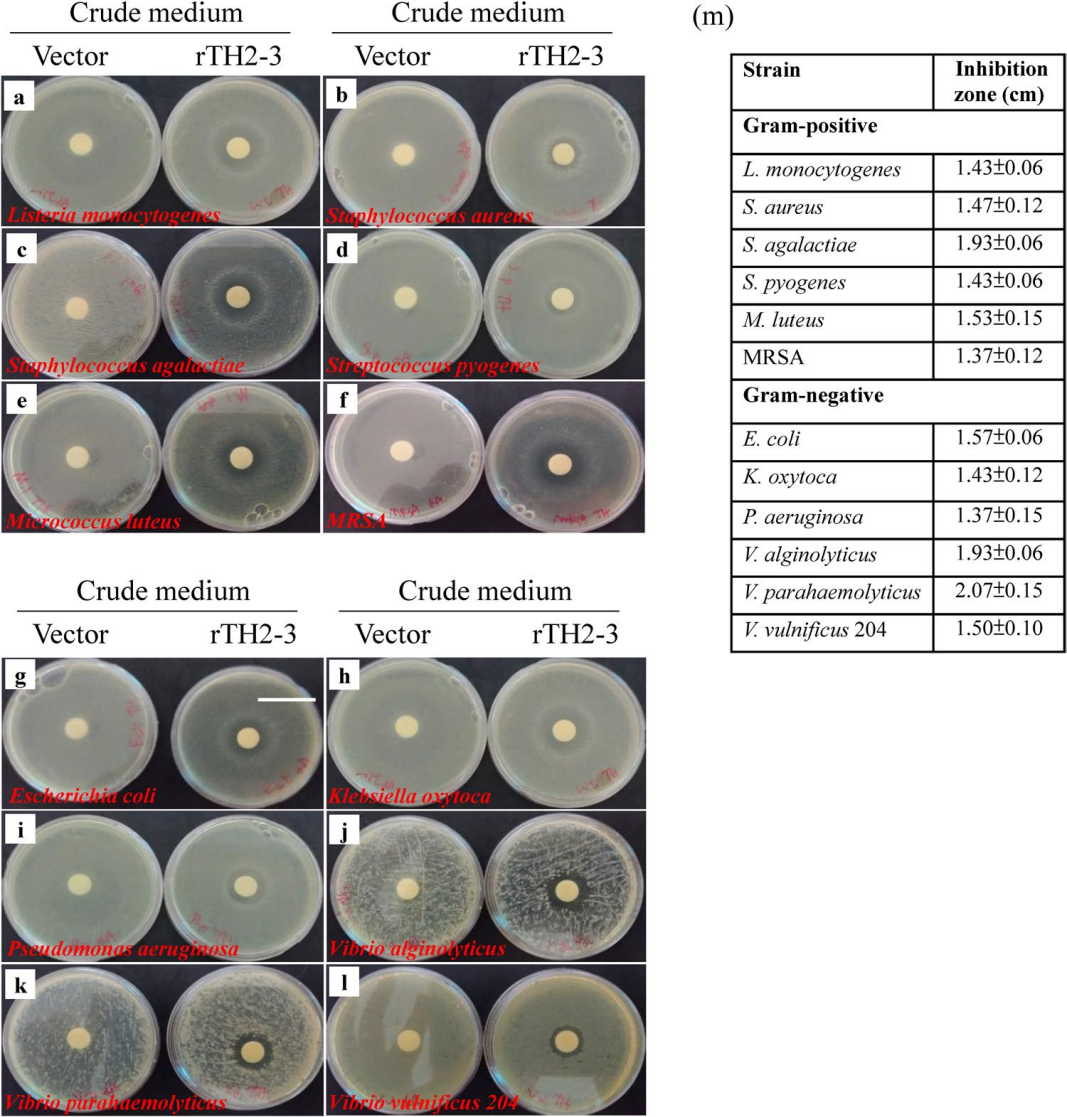
rTH2-3 shows broad-spectrum antimicrobial activity. (**a**–**l**) Eight-times concentrated supernatants from both methanol-induced Vector control and rTH2-3 expressed *P. pastoris* were used to test antibacterial activity by the disk diffusion assay. Six Gram-positive bacterial strains, including *Listeria monocytogenes* (**a**), *Staphylococcus aureus* (**b**), *Staphylococcus agalactiae* (**c**), *Streptococcus pyogenes* (**d**), *Micrococcus luteus* (**e**), *MRSA* (**f**) and six Gram-negative bacterial strains, including the *Escherichia coli* (**g**), *Klebsiella oxytoca* (**h**), *Pseudomonas aeruginosa* (**i**), *Vibrio alginolyticus* (**j**), *Vibrio parahaemolyticus* (**k**), *Vibrio vulnificus 204* (**l**) were evaluated. Bar: 4 cm. (**m**) The inhibition zones presented in (**a**–**l**) are representative of three independent assays. Results are presented as mean ± SD.

**Figure 4:**
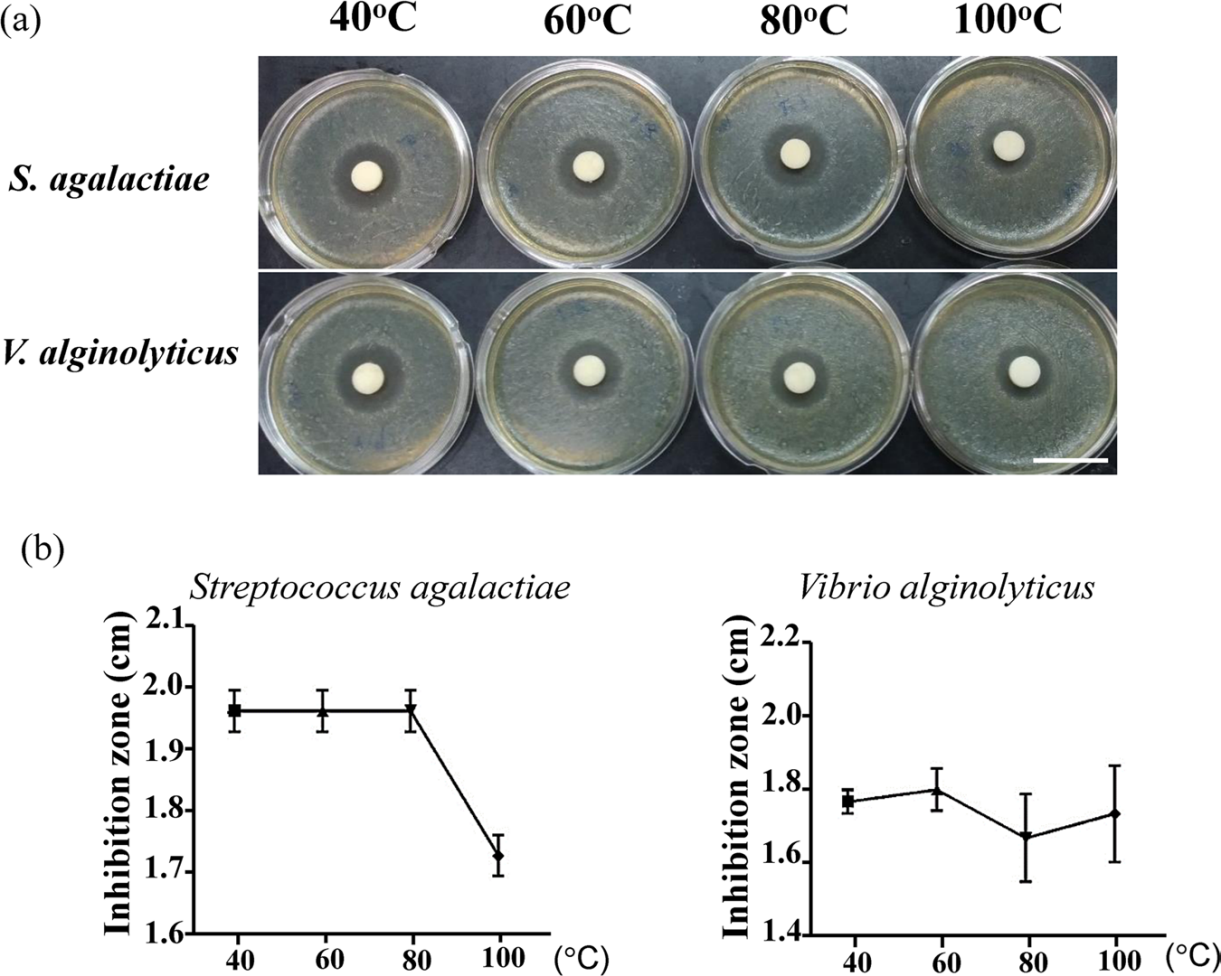
Thermal stability of rTH2-3. (**a**) Eight-times concentrated supernatant from methanol-induced *P. pastoris* was incubated at indicated temperatures and then used to assess antibacterial activity against *Staphylococcus agalactiae* (upper panel) and *Vibrio alginolyticus* (lower panel) by the disk diffusion assay. Bar: 4 cm. (**b**) The inhibition zones presented in (**a**) are representative of three independent assays. Results are presented as mean ± SD.

To evaluate whether yeast-derived rTH2-3 has antimicrobial activity, crude medium from the fermenter culture was collected and analyzed in a disk diffusion assay. Six Gram-positive bacteria strains (*L. monocytogenes, S. aureus, S. agalactiae, S. pyogenes, M. luteus, MRSA*) (Fig. 3a–f) and six Gram-negative bacteria strains (*E. coli, K. oxytoca, P. aeruginosa, V. alginolyticus, V. parahaemolyticus, V. vulnificus* 204) (Fig. 3g–l) were used to test the antimicrobial ability of rTH2-3. According to this assay, rTH2-3 harbored broad-spectrum antimicrobial activity against all tested bacteria strains (Fig. 3m). To further test the thermal stability of rTH2-3, concentrated crude medium was incubated at different temperatures (40–100 °C) prior to evaluating the antimicrobial activity by the disk diffusion assay (Fig. 4a). Even after incubation with high temperatures, rTH2-3 retained antimicrobial ability against both *S. agalactiae* and *V. alginolyticus*, indicating that it is thermally stable (Fig. 4b).

### Feeding strategy and growth performance

**Figure 5:**
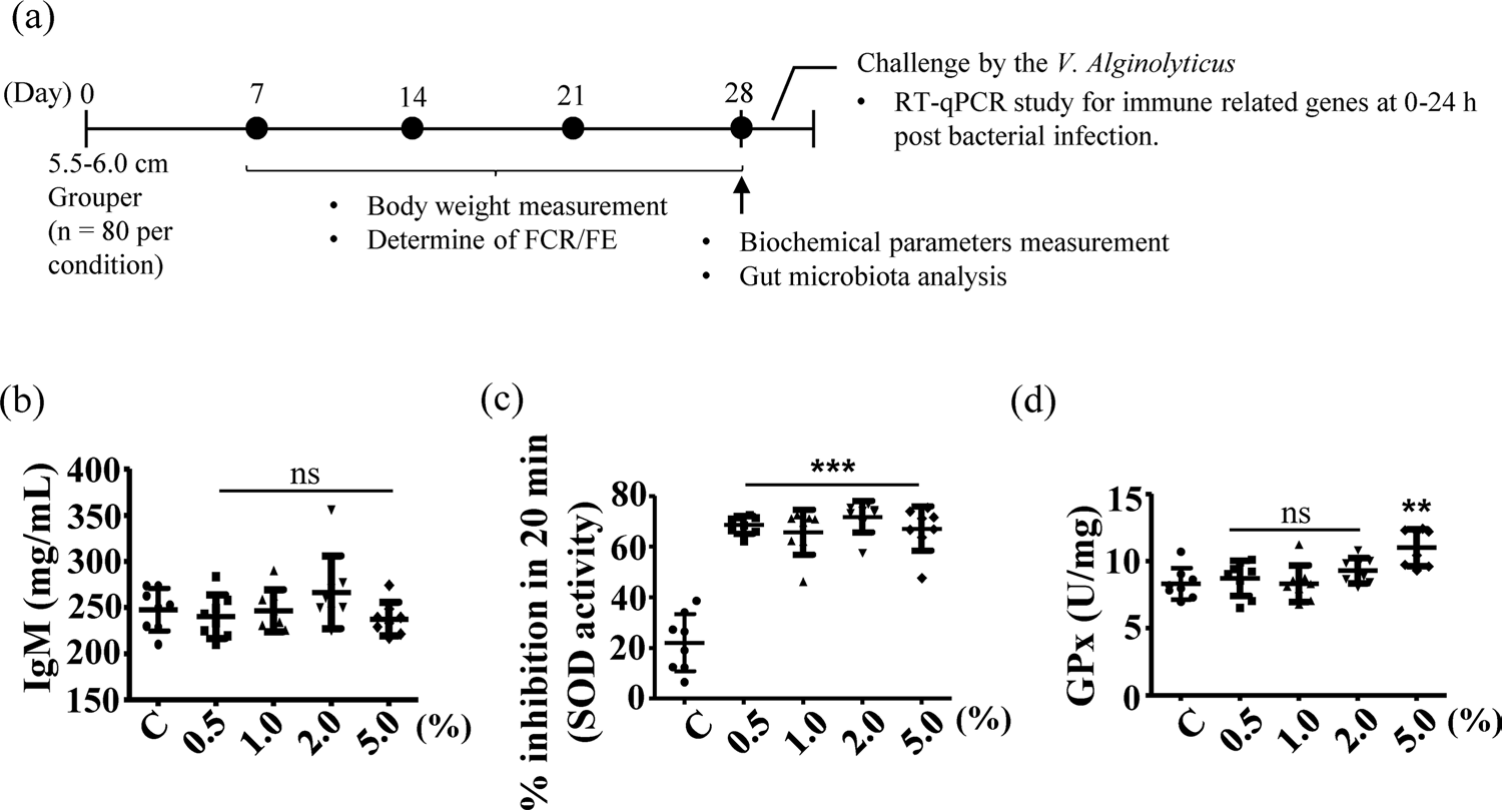
Biochemical parameters in rTH2-3-supplemented grouper. (**a**) The feeding scheme and analyses used in this study. (**b**–**d**) Levels of IgM (**b**), SOD (**c**), and GPx (**d**) were quantitatively measured in serum samples from fish (n = 8 per group), as determined by ELISA. Results are presented as mean ± SD, ns: not significant, **p < 0.01; ***p < 0.001 between groups by one-way ANOVA.

To assess the effects of rTH2-3-supplemented feed on the growth performance of grouper, larvae with a mean body length of 5.5–6.0 cm were randomly assigned to regular-diet-feeding and 0.5–5.0% rTH2-3 supplemented-diet-feeding groups (n = 80 per group; four tanks were used with 20 fish in each tank for each experimental group). Fish were fed with the rTH2-3-supplemented feed twice daily in the morning and evening, and an additional feeding with regular feed was provided in the afternoon. The compositions of regular and rTH2-3 diets are shown in Table 1. Body weights were measured every seven days over the course of 28 days (Fig. 5a). Groupers given rTH2-3-supplemented feed exhibited feed conversion ratios (FCR) ranging from 0.65 to 0.71, and the control group exhibited an FCR of 0.97. The feed efficiencies (FE) in the rTH2-3-diet-fed groups were 1.4–1.52, compared to 1.03 in the control group (Table 2). To assess the physiological effects of daily rTH2-3 administration, we determined the immunoglobin M (IgM), superoxidase dismutase (SOD), and glutathione peroxidase (GPx) levels by enzyme-linked immunosorbent assays (ELISAs). The IgM level was measured in blood samples collected from the control and rTH2-3-diet-fed groups. The results showed no significant differences in IgM values between groups (Fig. 5b). However, a significant enhancement of hepatic SOD activity was observed in the 0.5–5% rTH2-3-fed groups compared to the regular-diet-fed group (Fig. 5c). In addition, increased hepatic GPx activity was observed in the liver of the 5% rTH2-3-supplemented group compared to the control (Fig. 5d).

**Table 1:** Composition of diets used in this study.

Ingredients	Normal	0.5	1.0	2	5
Content (g/100 g feed)					
Fish meal	40	40	40	40	40
Squid liver meal	5	5	5	5	5
Soy flour	23	22.5	22	21	18
Yeast	4	4	4	4	4
Flour	12	12	12	12	12
Fish oil	3	3	3	3	3
Vitamin premix	5	5	5	5	5
Mineral premix	3	3	3	3	3
Starch	5	5	5	5	5
Additive (rTH2-3)	0	0.5	1	2	5
**Proximate analysis composition**					
Crude protein	39.28	38.94	39.4	39.8	40.42
Crude lipid	13.30	13.23	13.52	12.78	12.19
Ash	15.76	15.72	15.99	15.30	15.82
Moisture	4.11	4.83	4.84	4.83	4.92

**Table 2:** Effect of Feed Additives on Growth Performance of *Epinephelus lanceolatus*. Results represent mean ± SD (n = 80 per experimental group).

Parameter/rTH2-3 (%)	0	0.5	1	2	5	MSE
Initial weight	6.22 ± 0.70	n/a	n/a	n/a	n/a	
7 Days weight (g)	9.34 ± 1.41	11.53 ± 1.29^a^	11.39 ± 1.88^b^	11.82 ± 1.59^a^	11.36 ± 1.33^c^	2.35
14 Days weight (g)	16.45 ± 2.03	16.64 ± 2.13 ^ns^	17.15 ± 2.59 ^ns^	17.69 ± 2.21 ^ns^	17.00 ± 3.21 ^ns^	6.06
21 Days weight (g)	22.09 ± 2.05	21.16 ± 2.60 ^ns^	22.08 ± 2.68 ^ns^	24.08 ± 2.63 ^ns^	23.10 ± 3.33 ^ns^	7.14
28 Days weight (g)	26.70 ± 3.78	29.22 ± 3.41^d^	30.30 ± 3.63^e^	30.02 ± 3.26 ^f^	27.66 ± 3.72 ^ns^	12.57
Feed conversion ratio (FCR)	0.97	0.68	0.65	0.66	0.71	
Feed efficiency (FE)	1.03	1.47	1.52	1.51	1.4	

Data were analyzed by one-way *ANOVA*. ^a^*p* < 0.0001; ^b^*p* = 0.0002; ^c^*p* = 0.0009; ^d^*p* = 0.0278; ^e^*p* = 0.0019; ^f^*p* = 0.0041 vs control group; ns: not significant. n/a: not available. MSE: mean squared error.

### Gut microbiota analysis

**Figure 6:**
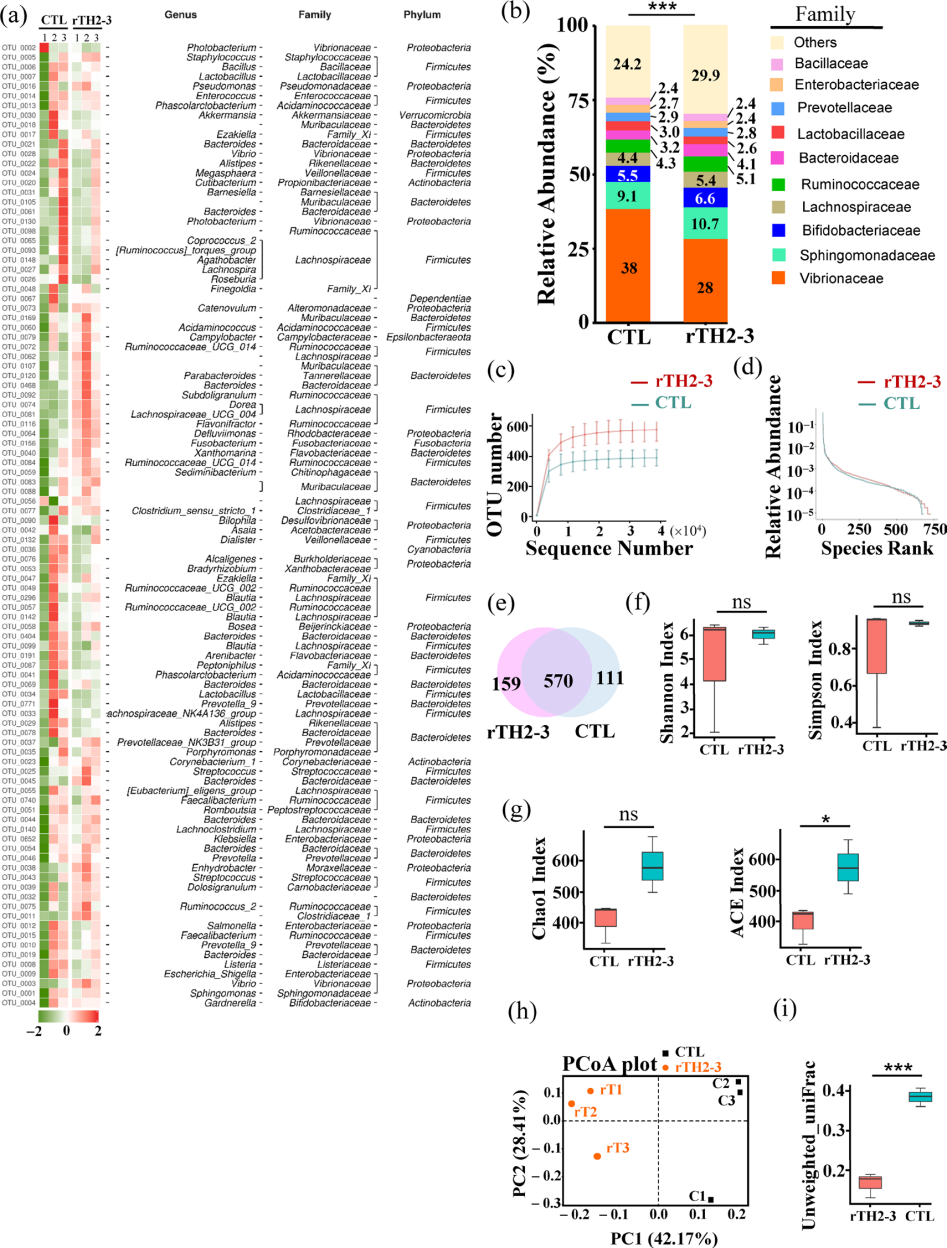
Gut microbiota profiling in the rTH2-3-fed grouper. (**a**) Heatmap shows the relative abundance of specific OTUs across samples (n = 3 in each group). Right panel illustrates the classification of each OTU at the genus, family, and phylum levels. (**b**) Top 10 relative abundance of taxon enrichment across samples. Statistical comparison between groups was performed by the Kruskal-Wallis test with *p* = 0.0005. (**c**) Rarefaction curves (Y-axis indicates number of observed species) for rTH2-3 diet versus regular diet. (**d**) The rank-abundance curve for all OTUs shared across samples. (**e**) The Venn diagram depicts the number of shared OTUs across groups. Numbers in non-overlapping regions indicate OTUs specific to individual groups. (**f,g**) Alpha-diversity was measured by Shannon’s and Simpson’s indexes (**f**) as well as Chao1 and ACE index (**g**). Box plots (showing the interquartile range) and the median values (black lines within boxes) are shown. Significant differences in alpha diversities between CTL and rTH2-3 diet are indicated (*p < 0.05, *t*-test; ns: not significant). (**h,i**) Beta-diversity was determined by PCoA analysis of unweighted UniFrac distance. Each dot labeled in the blot indicates one sample from the regular diet (CTL) or rTH2-3 diet group (**h**). Beta-diversity index difference based on unweighted UniFrac is shown (**i**). ***p < 0.001, *t*-test.

**Figure 7:**
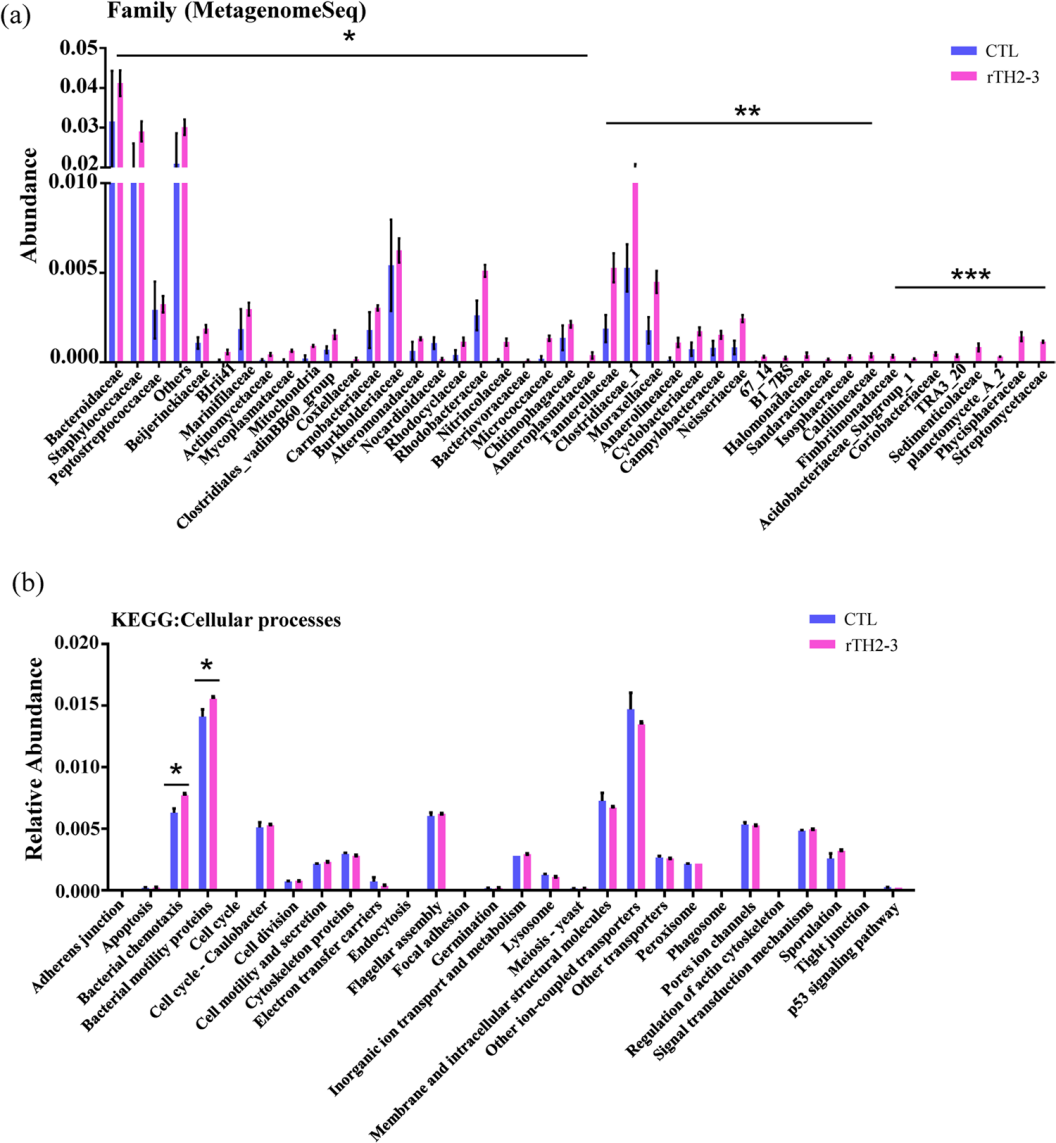
Metagenomic functional prediction of the gut microbiota in the grouper supplemented with dietary rTH2-3. (**a**) Abundance analyses between groups at the family level with significant differences. Results are presented as mean ± SEM (n = 3 in each group), *p < 0.05; **p < 0.01; ***p < 0.001 between groups, analyzed by STAMP. (**b**) PICRUSt classification of KEGG Orthology (KO). Predicted functional genes in the ‘cellular processes’ category of the gut microbiota are shown at KEGG level 3. Results are presented as mean ± SEM (n = 3 in each group), *p < 0.05 between groups by t-test.

We subsequently asked whether grouper fed with rTH2-3-supplemented diet may harbor a gut microbiota composition that is different from control fish, as suggested by an earlier report that showed expression of exogenous Hepcidin influenced the gut bacterial community^23^. The foregut from groupers fed with 1% rTH2-3-supplemented diet and controls (n = 3) were prepared for DNA extraction, and microbial profiling was conducted by the 16S amplicon sequencing method. The 1% rTH2-3-diet-fed group was selected because it exhibited the best FCR/FE value. The heatmap in Fig. 6a shows the relative abundance of gut-microbial constituents at the genus, family, and phylum levels. Significant differences in the relative microbial abundance between rTH2-3-supplemented-diet and control groups included a decrease in the family *Vibrionaceae* (38% in control-diet group and 28% in the rTH2-3-supplemented group) and increases in the families *Bifidobacteriaceae* (5.5% in control-diet group and 6.6% in the rTH2-3-supplemented group), *Lachnospiraceae* (4.4% in control-diet group and 5.4% in the rTH2-3-supplemented group), *Ruminococcaceae* (4.3% in control-diet group and 5.1% in the rTH2-3-supplemented group), and *Bacteroidaceae* (3.2% in control-diet group and 4.1% in the rTH2-3-supplemented group) (Kruskal-Wallis test, *P* = 0.0005, Fig. 6b). It has been reported that short-chain fatty acids (SCFAs) (*i.e*. butyrate, acetate, and propionate) are mainly produced by the phyla *Firmicutes* and *Bacteroidetes*^30^. At the Genus level, we observed that 33 out of 67 (~49.3%) bacteria species showing significant differences in abundance between groups belonged to the *Firmicutes* and *Bacteroidetes* phyla (Supplementary Fig. 1a,b and dataset 1). Most butyrate producers in the *Firmicutes* phylum belong to the clostridial clusters IV (family *Lachnospiraceae*) and XIVa (family *Ruminococcaceae*). Our data revealed that 16 out of 17 bacteria belonging to these families had increased abundance in the rTH2-3 supplementation group, with the exception of genus *Lachnospiraceae_UCG_010* (Supplementary Fig. 1a). Another eight bacteria belonging to the *Bacteroidetes* phylum had increased abundance in the TH2-3-supplemented group compared to regular diet group (Supplementary Fig. 1b). In addition, the rarefaction curve and rank abundance curve, which reflect species richness and biodiversity, differed between groups, with a much broader microbial population identified in the rTH2-3-supplemented-diet group (Fig. 6c,d). A Venn diagram illustrates that a total 570 operational taxonomic units (OTUs) were found in both groups, while 159 and 111 unique OTUs existed in rTH2-3-supplemented and control groups, respectively (Fig. 6e). We further assessed the richness, evenness, and diversity according to the Shannon-diversity index, Simpson index, Chao1-diversity index, and Abundance-based Coverage Estimator (ACE-diversity index). There were no significant differences between groups in terms of the Shannon and Simpson indexes (Fig. 6f). The Chao1 index showed a trend toward increase in the rTH2-3-supplemented group, while the ACE-diversity index was significantly higher in the rTH2-3 group compared to the regular diet (control) group (Fig. 6g, *p* < 0.05). An unweighted principal component analysis (unifrac PCoA) showed that the gut microbial compositions were different among the regular (CTL) and rTH2-3 diet groups. The microbiota in the rTH2-3-supplemented group clustered along the principal coordinates (PC) 1 and 2 and were separated from those in the CTL group (Fig. 6h). In addition, with the exception of rT3 and C1, the samples in each group clustered separately, indicating that the differences in the microbial communities within the groups were small, whereas rT3 and C1 were specific. The rTH2-3 group had significantly greater PCoA variation than the CTL group (Fig. 6i, *p* < 0.001). Furthermore, statistical analysis comparing the microbial communities of the two groups was performed with MetagenomeSeq (STAMP method, Welch’s t-test), and the results are shown in Fig. 7a. We also used these data to compare metabolic pathways in the microbial community with the KEGG database. Among six metabolic pathways (cellular processes, environmental information processing, genetic information processing, human diseases, metabolism, organismal systems), the ‘cellular processes’ pathway contained two categories (‘bacterial chemotaxis’ and ‘bacterial motility proteins’) that were significantly increased in the rTH2-3-supplemented-diet group compared to the regular-diet group (Fig. 7b and Supplementary Fig. 2a–g).

### Bacterial challenge and analysis of immune gene expression

**Figure 8:**
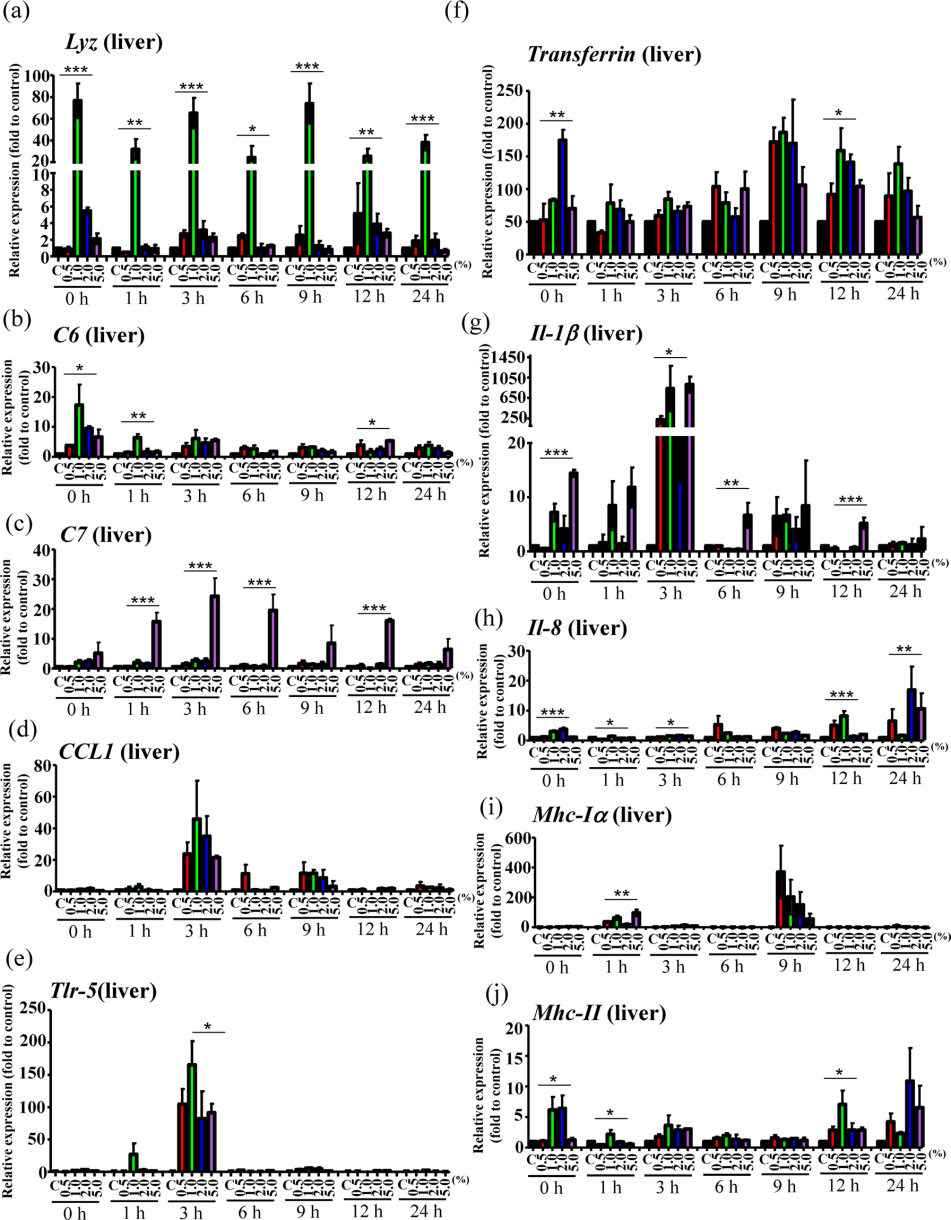
Hepatic expression patterns of immune genes in grouper supplemented with rTH2-3. (**a**–**j**) Relative expression of immune-related genes in the liver from grouper after challenge by *V. alginolyticus*. Immune-related gene expression, including *Lyz* (**a**), *C6* (**b**), *C7* (**c**), *CCL1* (**d**), *Tlr-5* (**e**), *Transferrin* (**f**), *Il-1β* (**g**), *Il-8* (**h**), *Mhc Iα* (**i**), and *Mhc II* (**j**), in fish fed with normal or rTH2-3-supplemented feed was determined by RT-qPCR. Fish were sacrificed at 0-24 h post-bacterial infection. Results are presented as mean ± SEM (n = 3 in each group), *p < 0.05; **p < 0.01; ***p < 0.001 between groups by one-way ANOVA.

**Figure 9:**
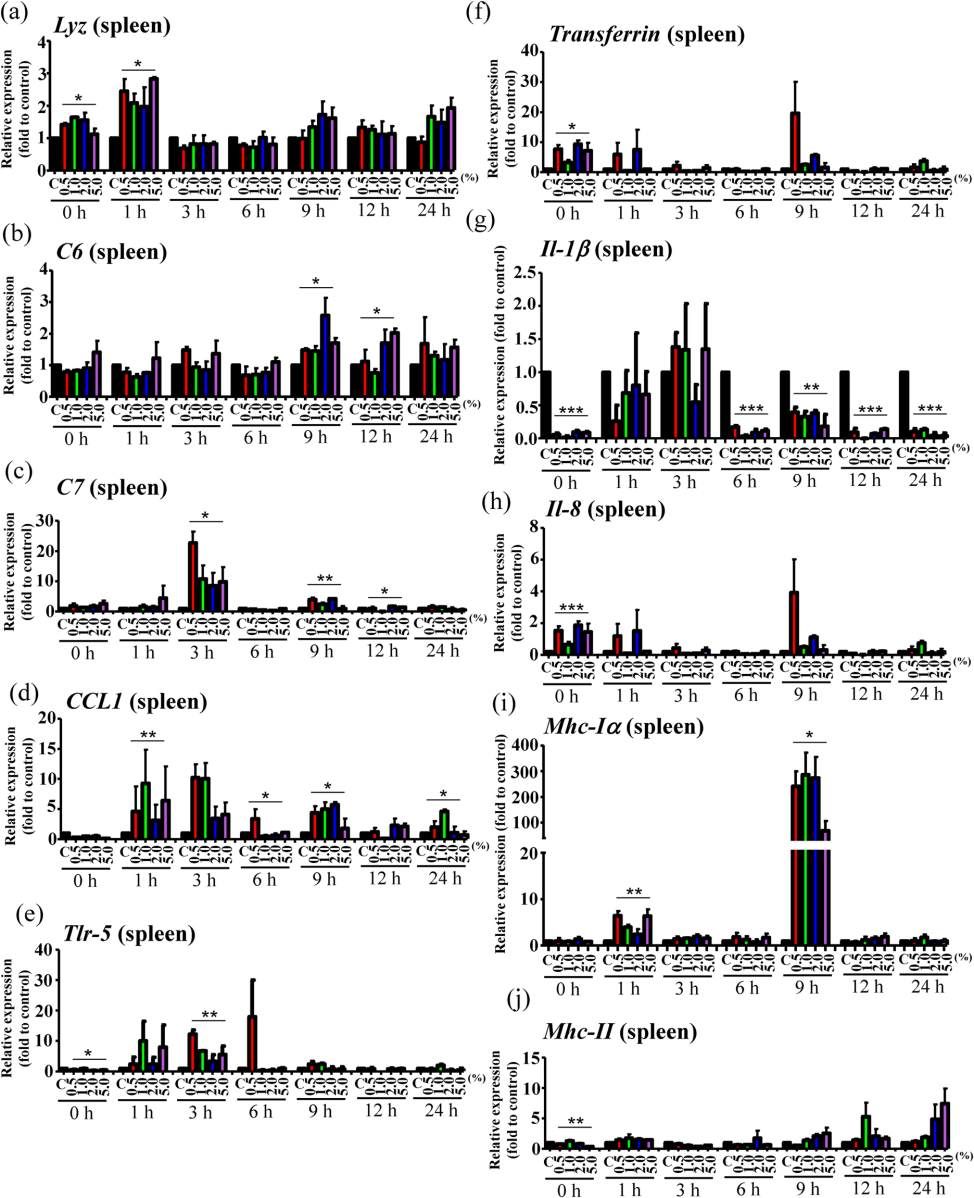
Splenetic expression patterns of immune genes in grouper supplemented with rTH2-3. (**a**–**j**) Relative expression of immune-related genes in the spleen from grouper after challenge by *V. alginolyticus*. Immune-related gene expression, including *Lyz* (**a**), *C6* (**b**), *C7* (**c**), *CCL1* (**d**), *Tlr-5* (**e**), *Transferrin* (**f**), *Il-1β* (**g**), *Il-8* (**h**), *Mhc Iα* (**i**), and *Mhc II* (**j**), in fish fed with normal or rTH2-3-supplemented feed was determined by RT-qPCR. Fish were sacrificed at 0-24 h post-bacterial infection. Results are presented as mean ± SEM (n = 3 in each group), *p < 0.05; **p < 0.01; ***p < 0.001 between groups by one-way ANOVA.

Since variations in gut microbiota composition may affect the immune system, we further evaluated whether grouper fed with rTH2-3-supplemented diet exhibit altered expression of immune-related genes in response to bacterial challenge. Fish were randomly selected for *V. alginolyticus* infection and were sacrificed at 0, 1, 3, 6, 9, 12, 24 h post-bacterial infection (n = 3 at each time point). Hepatic/splenetic tissues were harvested for RT-qPCR analysis of immune-associated genes (Figs. 8 and 9). In the hepatic tissue, *Lysozyme*, *Complement component 6* (*C6)*, *C7*, *Transferrin*, *Interleukin* (*Il)-1β*, *Il-8* and *Major histocompatibility complex (Mhc)-II* genes were obviously affected (Fig. 8a–c,f–j); *Toll-like receptor (Tlr-5)* and *Mhc-Iα* were mildly affected (Fig. 8e,i), and *C-C chemokine ligand-1* (*CCL1*) was not significantly affected (Fig. 8d) by the supplemented diet. In the splenetic tissue, *Lysozyme*, C6, *C7*, *CC chemokine-1*, *Tlr-5, Il-1β* and *Mhc-1α* genes were obviously affected (Fig. 9a–e,g and i), while *Transferrin*, *Il-8* and *Mhc-II* were mildly affected (Fig. 9f,h and j).

## Discussion

In the present work, we show that the use of rTH2-3 as a dietary feed supplement for grouper may impact growth performance (Table 2) and increased gut microbiota diversity (Figs. 6c–i and 7a, and Supplementary Fig. 1). Additionally, we observed an increase of hepatic SOD activity in all rTH2-3-supplemented groups, suggesting improved resistance to oxidative stress that may arise from bacterial infections. Moreover, KEGG analysis revealed a significant enrichment of bacterial chemotaxis and motility-related genes (Fig. 7b). It has been reported that one third of all underrepresented functions in gut bacteria are involved with motility and chemotaxis^31^, and these functions are correlated with the composition and metabolic activities of bacterial communities^32^. Thus, our findings suggest enhanced metabolic activity and alterations in the gut bacterial community are beneficial to rTH2-3-fed grouper.

During the process of air drying rTH2-3, the inlet air temperature reached 110 °C. Therefore, whether AMP activity may be diminished by heating is an important concern in our study. In our *in vitro* heating experiment (Fig. 4), we observed that TH2-3 heated to 100 °C still retained antibacterial activity, however, the activity was reduced compared to non-heated samples (Fig. 4b). Importantly, supplementation of rTH2-3 produced with the high-temperature spray dry method was still effective, and the supplement showed biological activity in the grouper. These results suggest that recombinant TH2-3 is quite stable during heating with only partial loss of bioactivity. A low-temperature dryer may be particularly suitable for biomass and fermented substances. Thus, low-temperature drying should be considered for the industrial application of large-scale-produced peptide feed additives.

Exogenously expressed recombinant Hepcidin derived from large yellow croaker (*Pseudosciaena crocea*) was previously used as a food additive; this additive influenced gut microbiota without causing inflammation^23^. Here we observed that feed supplemented with tilapia-derived Hepcidin has similar effects on intestinal bacterial composition in grouper (Fig. 6). Gut microbiota diversity is believed to be intimately linked with health and disease. Higher diversity has been proposed to confer higher resilience within small microbial systems and is associated with improved health status^33^. Under cellular stresses, such as oxidative stress from severe acute malnutrition, oxidative stress-sensitive anaerobes and methanogens are depleted, while oxidative stress-resistant aerotolerant bacterial pathogens are enriched in parallel, suggesting that imbalances in the intestinal redox status are associated with microbiota alterations^34^. Daily dietary antioxidant supplements have been shown to control oxidative stress and increase *Lactobacillus* and *Bifidobacterium* (potential beneficial microbes) counts, while decreasing *E. coli* counts in piglet^35,36^. An obvious difference between the rTH2-3-supplemented and control groups is the decreased relative abundance of *Vibrionaceae* (28% compared to 38% in the group fed with regular diet), which is resistant to oxidative stress. Conversely, increases were observed in the families *Bifidobacteriaceae* (6.6% compared to 5.5% in the group fed with regular diet), *Lachnospiraceae* (5.4% compared to 4.4% in the group fed with regular diet), *Ruminococcaceae* (5.1% compared to 4.4% in the group fed with regular diet), and *Bacteroidaceae* (4.1% compared to 3.2% in the group fed with regular diet), which are sensitive to oxidative stress (Fig. 6b). The family *Vibrionaceae* belongs to the proteobacteria phylum, which is associated with a high Immunoglobin A (IgA) response^37^ and includes several pathogenic species with broad host susceptibility. IgA secretion from the mucous membrane is the main Ig response that is induced by the presence of commensal microbes in the intestine, and this Ig functions in immune clearance through highly specific binding to bacterial outer cell wall antigens^38^. The family *Vibrionaceae*, which includes *V. Cholerae*, is able to evade the host immune system by turning off the biosynthesis of IgA-targeted mannose-sensitive hemagglutinin (MSHA) through activation of the virulence transcription factor *ToxT*, and by doing so, it may successfully colonize the intestine^39^. The abundance of such oxidative stress-resistant bacteria (*i.e*. proteobacteria) triggers pro-inflammatory immune responses associated with excessive oxidative stress; meanwhile, the maintenance of existing minor populations of oxidative stress-sensitive bacteria is crucial for intestinal immune homeostasis^38^. For example, health-promoting bifidobacteria are highly susceptible to acid and oxidative stresses^40^. *B. adolescentis* is able to evade T-cell-independent IgA and evoke T-cell-dependent immune responses^41,42^. Additionally, the families *Lachnospiraceae*, *Ruminococcaceae* and *Bacteroidetes* are gut bacteria that produce SCFAs, a major regulator of gut mucosal immunity (particularly in the terminal ileum and colon) that serves as a marker of a healthy and mature population of anaerobic gut microbiota (HMAGM)^43,44^. The SCFA, butyrate, is known as a histone deacetylase inhibitor^45^, which functions to downregulate gut mucosal immunity by increasing FoxP3 + T regulatory (Treg) cells within the colon^46^ and negatively regulating NKp46 + group 3 innate lymphoid cells (ILC3s) in terminal ileal Peyer’s patches to induce antigen-specific immunity^47^. Dietary supplementation of sodium butyrate (2000 mg kg^−1^) improved growth, antioxidant capacity and intestinal absorption in the juvenile grass carp (*Ctenopharyngodon idellus*)^48^. Partially protective effects on growth performance and intestinal metabolism were also reported in sea bream (*Sparus aurata*) that received dietary butyrate supplementation^49^. In a rat model of insulin resistance and steatosis induced by high-fat diet, administration of butyrate reduced liver steatosis and inflammation^50^. In addition, a high-fiber diet and acetate supplementation led to changes in the gut microbiota and improved cardiovascular health and function in hypertensive mice^51^. These results suggest SCFA supplementation improves gut microbial composition to promote health status or mitigate disease. We found that potential SCFA-producing bacteria (including 24 *Lachnospiraceae* and *Ruminococcaceae* families and 8 *Bacteroidetes* family microbes) were more abundant in the rTH2-3 diet group than controls, accounting for nearly half of affected microbes (Supplementary Fig. 1 and dataset 1). This finding suggests that these SCFA-producing families of bacteria, which differentially regulate gut immunity and are critical to maintain the health of the gut, were enhanced in grouper by rTH2-3 dietary supplementation.

In addition, enhanced immunity was observed in the liver and spleen of *V. alginolyticus*-infected grouper fed with rTH2-3-supplemented diet, when compared to those fed with regular diet. This enhancement may have been due to the production of certain SCFAs that promote systemic immunity^52–54^. *Lyz*-encoded Lysozyme functions as an AMP that is involved in host-defense mechanisms and immune modulation^55^. Lysozyme catalyzes the hydrolysis of peptidoglycans on Gram-positive bacteria and subsequently causes bacterial cell lysis through an AMP-mediated lytic mechanism^56–58^. In addition, lysozyme can attack Gram-negative bacteria^58–60^. Robust *Lyz* activation was observed in the liver (and at 0 and 1 h post-infection in the spleen) of bacteria-infected grouper (Figs. 8a and 9a). However, the liver regulates innate defense mechanisms, and a major induction of lysozyme expression upon stress should be detected in numerous tissues, including liver, secretory immune cells^61^ and the mucosal surface^62,63^. Therefore, lysozyme may have been systemically activated in response to bacterial infection in grouper, with its expression not limited to the liver. In addition, a trend toward upregulation of *transferrin* expression was observed at 9–24 h post-bacterial infection (Fig. 8f). Transferrin acts as a component of innate immunity by sequestering iron, reducing its availability to invading pathogens^64,65^. While Transferrin level is normally decreased under inflammatory conditions^66^, enhanced Transferrin level may reflect in improved status for bacteria clearance as well as tissue recovery; this interpretation is also supported by the observed decrease of pro-inflammatory cytokines *Il-1β* and *Il-8* (Figs. 8g,9g,h). In addition, significant *Tlr-5* upregulation was found prior to 6 h post-bacterial infection (Figs. 8e and 9e). Tlr-5 regulates colonization of gut microbiota to prevent inflammation^67^ and thus may serve as an indicator of attenuated pro-inflammatory processes.

The membrane attack complex (MAC) is composed of C5b, C6, C7, C8 and several C9 molecules and is assembled in response to complement activation^68^. The MAC complex functions to disrupt the cell membrane of targeted microbes, leading to cell death. Increased expression of hepatic and splenetic *C6* and *C7* was observed in the rTH2-3-supplemented-diet group compared to control fish upon bacterial infection (Figs. 8b,c and 9b,c), suggesting the Complement system was activated in supplement-fed fish to enhance bacterial killing. Additionally, a strong trend toward increase of *MHC-I* expression was observed at 9 h post-bacterial infection in the rTH2-3-supplemented fish compared controls (Figs. 8j and 9j), indicating an activation of both cytotoxic and CD8 + suppressor T-lymphocyte host-defense mechanisms that together reflect overall immune function (*i.e*. CD4 + T-lymphocytes activation, phagocytes recruitment, and B cell activation)^69,70^. Interestingly, enhanced *CCL1* activation was found in the spleen but not in the liver of grouper supplemented with rTH2-3 (Figs. 8d and 9d). In fish, the spleen is the organ centrally responsible for regulating immune system function in response to pathogen infection, via its promotion of lymphocyte maturation^71^. *CCL1* encodes C-C chemokine ligand 1 (CCL1) which is secreted by activated T-cells, monocytes and mast cells. The CCL1 protein binds to its cognate receptor, Chemokine (C-C motif) receptor 8 (CCR8), which is expressed in circulating CCR8 + regulatory T cells (Treg cells), interstitial dendritic cells, Langerhans cells and their monocytic precursors^72^. The production of CCL1 by Treg cells upregulates the expression of CCR8 to promote the immunosuppressive actions of these cells^73^, suggesting that regulation of the CCL1-CCR8 axis in grouper prevents excessive immune response after infection.

Our study shows that dietary supplementation with 1% rTH2-3 enhanced health status and intestinal microbial diversity in grouper, which may protect from bacterial infection via immunomodulatory mechanisms. The yield of rTH2-3 was about 23.37 mg per g of spray-dried powder (23.37 mg/g), making the production cost about US$1.5 for 1 mg of rTH2-3. This cost is based on the use of commercial BMGY medium and may be lowered by replacing the BMGY medium with industrial basal salt medium. Our past experience in peptide production suggests that such a replacement would lower the cost of rTH2-3 production by at least 50%. Since we aim to further develop rTH2-3-supplemented functional feed to reduce the use of medicated feeds, a small-scale field test in an indoor farm will be conducted in the near future. In addition, rTH2-3 from *Pseudosciaena crocea* has also been used as a feed additive to improve bacterial resistance. Therefore, rTH2-3 and other AMPs may be highly useful as feed or food supplements in fisheries and animal husbandry.

## Methods

### Plasmid construction

The TH2-3 expression plasmid was constructed by whole gene synthesis with codon optimization (GenScript Biotech Corp.). The coding sequence of TH2-3 was fused in-frame with cDNA sequence encoding the α factor signal peptide at the 5′-end and a 6×His tag at the 3′-end. The vector backbone was pPICZα-A (ThermoFisher Scientific).

### Expression and purification of the recombinant protein

Before transformation, plasmid was linearized by *Sac* I. Linearized plasmid was transformed into X-33 *Pichia Pastoris* cells by electroporation (ECM 399 electroporation system, BTX Harvard apparatus). The optimized electroporation conditions were as follows: 1.5 kV, 25 μF, 200 Ω. A single colony of the electroporated cells was grown on a yeast-extract-peptone-dextrose-sorbitol (YPDS) plate with Zeocin (100 μg/ml) or a minimal methanol with histidine agar plate for the selection of cells with the Mut^+^ phenotype. Protocols were performed in accordance with the manufacturer’s recommendation (EasySelect™ Pichia Expression Kit, ThermoFisher Scientific). For large scale production of rTH2-3, cells were grown in YPD medium at 30 °C (250–300 rpm) for 24 h until the optical density (OD) reached 2–6. Then cells were moved to the fermenter (Winpact Parallel Fermentation System, Major Science, Saratoga, CA, USA) and cultured in Buffered Glycerol Complex (BMGY) medium. The conditions for the batch culture (*e.g*. temperature, agitation and aeration parameters, pH value) followed the manufacturer’s standard settings. The batch was cultured until the glycerol was completely consumed (19 h) followed by 50% w/v glycerol feeding for 6 h. Next, a 100% methanol feed was started and maintained for 24–96 h. Cultures were then centrifuged at 6000 rpm for 30 min and supernatants were spray-dried by using a spray dryer (YC-500 laboratory spray dryer, Shanghai Pilotech Instrument&Equipment Co., Ltd) for further feed production. The spray drying parameters were set with an inlet air temperature of 110 °C and outlet air temperature of 65 °C, respectively.

### Protein identification and Western blotting

Samples were harvested in Radio-Immunoprecipitation Assay (RIPA) buffer (20 mM Tris-HCl pH 7.4, 150 mM NaCl, 1 mM Na2-EDTA, 1 mM EGTA, 1% Igepal CA-630 [Sigma-Aldrich], 1% sodium deoxycholate, 2.5 mM sodium pyrophosphate, 1 mM β-glycerophosphate, 1 mM Na_3_VO_4_, protease inhibitor cocktail [Roche Applied Science]). Lysates were then electrophoresed and transferred onto PVDF membrane. For the LC-MS/MS analysis, boiled lysates were electrophoresed on 15% SDS-PAGE and gels were stained by InstantBlue^TM^ solution (Expedeon Ltd). Excised protein slices were processed for tryptic in-gel digestion and LC-MS/MS analysis (Q-Exactive LC-MS, Thermo Scientific). The MS data were analyzed by Mascot engine (v.2.6.0) to identify candidate proteins. For Western blot, membranes were incubated in blocking buffer (0.1 M PBS, 5% non-fat milk, 0.1% Tween-20) for 1 h at room temperature and then incubated in the same solution with 6×His antibody (1:5,000, ThermoFisher Scientific) and donkey anti-mouse secondary antibody (GE Healthcare Life Science). Signal was visualized with enhanced chemiluminescence (Immobilon Western Chemiluminescent HRP substrate, Merck Millipore) and detected by an imaging system (UVP, BioSpectrum^TM^ 500).

### Disk diffusion assay

Bacterial strains were purchased from the Bioresource Collection and Research Center (BCRC, Hsinchu, Taiwan) and the American Type Culture Collection (ATCC, Manassas, VA, USA) and cultured as recommended. Cells used in this study were: *Listeria monocytogenes* (BCRC14845), *Staphylococcus aureus* (BCRC10451), *Staphylococcus agalactiae* (BCRC10787T), *Streptococcus pyogenes* (BCRC10797), *Micrococcus luteus* (BCRC11034T), *Escherichia coli* (BCRC10675T), *Klebsiella oxytoca* (BCRC13985T), *Vibrio alginolyticus* (BCRC12829T), *Vibrio parahaemolyticus* (BCRC10806T), and *Pseudomonas aeruginosa* (ATCC19660). The methicillin-resistant Staphylococcus aureus (*MRSA*) and the *Vibrio vulnificus 204* were cultured as previously described^8,10^. The disk diffusion assay was performed as previously described^21^. Briefly, each bacterial culture was grown to an OD value of 1–1.5 and inoculated on a culture plate overnight. Eight-times concentrated crude medium (~50 μL) was added drop-wise to filter paper strips and then placed in the center of the culture plate that had been inoculated with bacterial strains. For the thermal stability assay, concentrated supernatant from rTH2-3-expressing cells were heated for 5 min at the indicated temperatures before further processing. We routinely applied a semi-quantitative method to determine the rTH2-3 level in each of the large-scale protein production batches. Briefly, spray-dried samples (200–300 mg) were dissolved in 1 mL distilled water. A total of 100 μL sample was then mixed with 20 μL 6× sample buffer and boiled at 100 °C for 15 min. Immunoblotting was then conducted to determine the rTH2-3 level. Synthetic TH2-3 peptide samples were probed on the same blot to create a standard curve that was analyzed by linear regression. Based on this approach, the average concentration of rTH2-3 from the fermenter was 23.37 ± 1.277 ng nL^−1^. The concentration of rTH2-3 used for the disk diffusion assay in this study was therefore determined to be an average of 9348 ng (=23.37 × 8 × 50) per 50 μL. The diffusion diameter of the inhibition zone size was recorded. Data were collected from three independent assays.

### *Epinephelus lanceolatus* aquaculture and feeding

All fish care and handing procedures were performed in accordance with Academia Sinica guidelines and “The Ethical Guideline for Using Vertebrates as Experimental Animals in Taiwan”. Experiments were approved by the “Ethical Committee for Using Vertebrates as Experimental Animals” of Academia Sinica (Protocol ID: 16-11-1002). *E. lanceolatus* larvae were purchased from the Department of Aquaculture, National Taiwan Ocean University. Grouper larvae were cultured in a customized fish farming tank (length × width × height: 50 × 50 × 76 cm; total volume: 190,000 cm^3^; operational volume: 123,525 cm^3^) and grown to 5.5–6.0 cm in length and 6.22 ± 0.70 g in weight. The fish farming tanks utilized a flow-through aquaculture system, where the water quality in each tank is independent of the others. To manage aquaculture water quality, sea water was first passed through a shallow medium filter and then sterilized with ozone. Treated water was transported into a reservoir with an aerator. Parameters of water quality, including the pH (8.22 ± 0.04), temperature (29.21 ± 0.60 °C), salinity (29.44 ± 0.53 ppt), and redox potential (512.50 ± 3.24 mV) were monitored daily using the iFISH system (Today’s Instruments Co., Ltd.) for real-time remote monitoring for aquaculture water quality. Water quality parameters during the experimental period are provided in Supplementary dataset 2. To assess the effects of rTH2-3-supplemented feed on the growth performance of grouper, larvae with a mean body length of 5.5–6.0 cm were randomly assigned to regular-diet, and 0.5–5.0% rTH2-3 supplemented-diet feeding groups (n = 80 per group, four tanks were used with 20 fish in each tank for each experimental group). The fish were fed three times daily (feed amount: 3% of fish body weight). Control and rTH2-3-supplemented feed was given at 09:30 and 16:30. All groups were given an additional feeding with control diet at 13:30. The fish weights were recorded initially and every seven days thereafter. After 28 days, fish weights from 80 fish were summed and used to calculate the overall feed conversion ratio (FCR) and feed efficiency (FE) in each experimental group. The feed conversion ratio (FCR) and feed efficiency (FE) were calculated after feeding for 28 days through the following formula: FCR = Total feed given (g)/Fish weight gain (g). FE = Fish weight gain (g)/Total feed given (g).

### Analysis of biochemical parameters

Serum or tissue homogenates (n = 8 per experimental groups) were used to measure IgM, SOD activity, and GPx activity with a Fish immunoglobulin M (IgM) ELISA Kit (CUSABIO TECHNOLOGY LLC, Houston, TX, USA), a Superoxide Dismutase Activity Assay Kit (Abnova cooperation, Taipei Taiwan), and a Glutathione Peroxidase Assay Kit (Colorimetric) (Abcam, Cambridge, UK), respectively. Standard procedures were followed in accordance with the manufacturer’s recommendations.

### Bacterial challenge, reverse transcription, and quantitative real-time PCR

Grouper were injected with *V. alginolyticus* (3.02 × 10^11^ CFU/fish in 80 μL) and sacrificed at 0, 1, 3, 6, 9, 12, 24 h after infection (n = 3 per group). We routinely used 50–100 μL bacteria for the bacterial challenge experiment in fish with a body length 10–11 cm. A high dose of bacteria (3.02 × 1011 CFU/fish) used in this study is because low toxicity was observed when this batch of bacteria was re-cultured from a glycerol stock. The mortality was about 30–40%; however, significant pathology was still observed (i.e. redness, pain, and tissue destruction) after V. alginolyticus infection. Liver and spleen were dissected for RNA extraction using TRIzol reagent (Thermo Fisher Scientific) in accordance with the manufacturer’s protocol. Total RNA (1 μg) was reverse transcribed, and quantitative PCR (qPCR) was performed with the StepOnePlus Real-Time PCR System (Applied Biosystems, Life technologies) as previously described^8^. The PCR conditions were as follows: 95 °C, 2 min (holding stage); 40 cycles of 95 °C, 10 sec, 60 °C, 5 sec, and 72 °C, 45 sec; 95 °C, 15 sec, 60 °C, 1 min, and 95 °C, 15 sec (Melting curve stage). The _ΔΔ_CT method was performed for gene expression analysis, as previously described^8^. The *Actb* gene was used as the calibrator. Specific primer sets for the immune-related genes were used based on previous transcriptome analysis^74,75^ and are listed in Supplementary Table 1.

### Gut microbiota analysis

The foregut from groupers fed with control or 1% rTH2-3 diet were dissected for genomic DNA extraction using innuSPEED Stool DNA kit (Thermo Fisher Scientific Inc., Analytic Jena, Göteborg, Sweden). Before tissue dissection, fish were starved for one day. A primer set (515F/806R) targeting the 16S rRNA gene sequence was used for PCR amplification with Phusion® High-Fidelity PCR Master Mix (New England Biolabs). PCR products between 400–450 bp were processed for DNA elution using a Qiagen Gel Extraction Kit (Qiagen, Germany). Sequencing libraries were generated using NEBNext® Ultra™ DNA Library Prep Kit for Illumina® in accordance with the manufacturer’s recommendations, and index codes were added. Quality of the library was assessed by the Qubit® 2.0 fluorometer (Thermo Fisher Scientific, Carlsbad, CA, USA) and Agilent Bioanalyzer 2100 system (Agilent Technologies, Inc.). The library was sequenced on an Illumina platform and 250-bp paired-end reads were generated. The FLASH tool (v.1.2.7) was used to merge pair-end reads^76^, and quality filtering by QIIME (v.1.7.0) was performed to obtain high quality tags^77^. Tags were then compared with the reference “Gold” database (http://drive5.com/uchime/uchime_download.html), and any chimeric sequence was removed by the UCHIME algorithm^78,79^. An operational taxonomic unit (OTU) was used to define a closely related individual. Sequences with ≥97% similarity as determined from Uparse software (v7.0.1001) were assigned to the same OTU^80^ and were processed for taxonomic annotation, based on the GreenGene Database_ENREF_78^81^ by the RDP classifier algorithm (Version 2.2)^82^. For the construction of phylogenetic relationships as well as dominant species among OTUs, multiple sequence alignments were performed using the MUSCLE software (Version 3.8.31)^83^. The OTU abundance was compared by normalization to a standard number of sequences corresponding to the sample with the fewest sequences, and this parameter was used to analyze the differences in diversity between groups. Alpha diversity was calculated according to diversity indexes (Shannon’s and Simpson’s diversity indexes) and species richness estimates (Chao1 and abundance-based coverage estimators [ACE] estimator)^84^. Beta diversity was measured by an unweighted UniFrac distance matrix through QIIME and Principal Coordinates Analyses (PCoA) on the distance matrixes^85,86^. The profiles of phylogenetic marker gene sequences (16S rRNA) were used to identify microbial communities as well as to predict the functional composition of a metagenome through a computational approach called the phylogenetic investigation of communities by reconstruction of unobserved states (PICRUSt) (V.1.1.0)^87,88^. MetagenomeSeq was used to statistically determine OTUs that were differentially abundant between groups with multiple hypothesis testing and FDR (False discovery rate)^89,90^. The taxonomic profiling and gene function annotation were predicted by the Kyoto Encyclopedia of Genes and Genomes (KEGG) orthologs. The KEGG pathways that aggregated to level 3 were further analyzed.

### Statistical analysis

Data from repeated assays (*n* ≥ 3) were analyzed by Prism 7 software (GraphPad Inc.). Values represent the mean ± mean of standard deviation (SEM) or standard deviation (SD), and the statistical significance of any difference was determined by applying a two-tailed *t*-test or one-way (*ANOVA*) with Tukey’s multiple comparison. The Mean squared error (MSE) was calculated for each *ANOVA* analysis. For the gut microbiota analysis, the species abundance between groups was analyzed by Kruskal-Wallis test or Welch’s t-test of the STatistical Analysis of Metagenomic Profiles (STAMP) method^91^. Differences were considered statistically significant at *P* < 0.05.

## Supplementary information

supplementary information

Supplementary dataset 1

Supplementary dataset 2

## Data Availability

The datasets in this study are available from the corresponding author upon reasonable request.
