# Supplementary material for: Impact of Tilapia hepcidin 2-3 dietary supplementation on the gut microbiota profile and immunomodulation in the grouper (Epinephelus lanceolatus)
Source: Sci Rep. 2019 Dec 13;9:19047. doi: 10.1038/s41598-019-55509-9 (PMC6910925; doi:10.1038/s41598-019-55509-9)

Additional Information

Impact of Tilapia hepcidin 2-3 dietary supplementation on the gut microbiota profile and immunomodulation in the grouper (*Epinephelus lanceolatus*)

Chen-Hung Ting [1], Chieh-Yu Pan [2], Yi-Chun Chen [1], Yu-Chun Lin [1], Tzong-Yueh Chen [3], Venugopal Rajanbabu[4], Jyh-Yih Chen [1,5,6*]

1. Marine Research Station, Institute of Cellular and Organismic Biology, Academia Sinica, 23-10 Dahuen Road, Jiaushi, Ilan 262, Taiwan
2. Department and Graduate Institute of Aquaculture, National Kaohsiung University of Science and Technology, Kaohsiung 811, Taiwan
3. Institute of Biotechnology & Department of Biotechnology and Bioindustry Sciences, National Cheng Kung University, Tainan 701, Taiwan
4. Anbil Dharmalingam Agricultural College and Research Institute, Tamil Nadu Agricultural university, Tiruchchirapalli 620027, Tamil Nadu, India
5. The iEGG and Animal Biotechnology Center, National Chung Hsing University, Taichung 402, Taiwan
6. Center of Excellence for the Oceans, National Taiwan Ocean University, Keelung 202, Taiwan

* Corresponding author (Jyh-Yih Chen)
Marine Research Station, Institute of Cellular and Organismic Biology, Academia Sinica, 23-10 Dahuen Road, Jiaushi, Ilan 262, Taiwan.

Tel.: +886-920802111
Fax: +886-39871035
E-mail address: zoocjy@gate.sinica.edu.tw

Supplementary Figure legends

Supplementary Figure 1. Abundance analyses at the genus level. **(a, b)** Bacteria from the phyla *Firmicutes* (a) and *Bacteroidetes* (b) were analyzed for their abundance in regular diet and rTH2-3-supplemented groups. Specific bacteria belonging to the families *Lachnospiraceae* and *Ruminococcaceae* are indicated in (a). Results are presented as mean ± SD (n = 3 in each group), $^{*}p < 0.05$; $^{**}p < 0.01$ between groups, analyzed by STAMP, Welch's t-test.

Supplementary Figure 2. PICRUSt classification of KEGG Orthology (KO). (**a–g**) Predicted functional genes in the categories of environmental information processing (a), genetic information (b), human diseases (c), immune system (d), metabolism (e–g) at KEGG level 3 are shown for the gut microbiota. Results are presented as mean ± SEM (n = 3 in each group).

Supplementary Table 1. Primer sequences used for quantitative real-time PCR.

Genes (Predicted)	Sequence	Reference species
β-Actin	F: TGTGTCATCTTCTCCCTGT	*Oryzias latipes*
	R: GAGAGGTATCCTGACTCTGAAGTA	
Lysozyme	F: GACATTCAGTCCAGCCTAGAGTCC	*Epinephelus coioides*
	R: CCCGACTCCCACTGGCTC	
C6	F: TTGAGGCGTCACAGCGA	*Amphiprion ocellaris*
	R: GGCCTGTTCTCTCAGGA	
C7	F: CGATCACAGCTCCAACC	*Labrus bergylta*
	R: AAGAGCCACAGAGGAGT	
CCL1	F: ATCTCAGTCTCAGGTTTCATTG	*Epinephelus coioides*
	R: TCTCCATCATCACATCCACT	
Tlr-5	F: AGAAATGGTGATTCAAGACCC	*Epinephelus bruneus*
	R: TCAAATGTGAATTTCCATCTGATTTCTAT	
Transferrin	F: GCAACATGGGCTAGTGGTGT	*Epinephelus coioides*
	R: AATGCGAACAACCTCATTGC	
Il-1β	F: TAAACCTCTGTGTGGCG	*Epinephelus coioides*
	R: CACTGACCTGCACAGAC	
Il-8	F: CATGATGGAAGCCATGAG	*Oplegnathus fasciatus*
	R: GTCAGCGTGTCAGGAAT	
Mhc-Iα	F: TCTCCTGGTGTTACTGTCATAG	*Epinephelus coioides*
	R: CGCTTCTGCTTCTCGTG	
Mhc-II	F: CATGGCCTCACCTCTAC	*Sebastes schlegelii*
	R: TGTGGGATTAGAACTGGACA	

Supplementary Figure 1

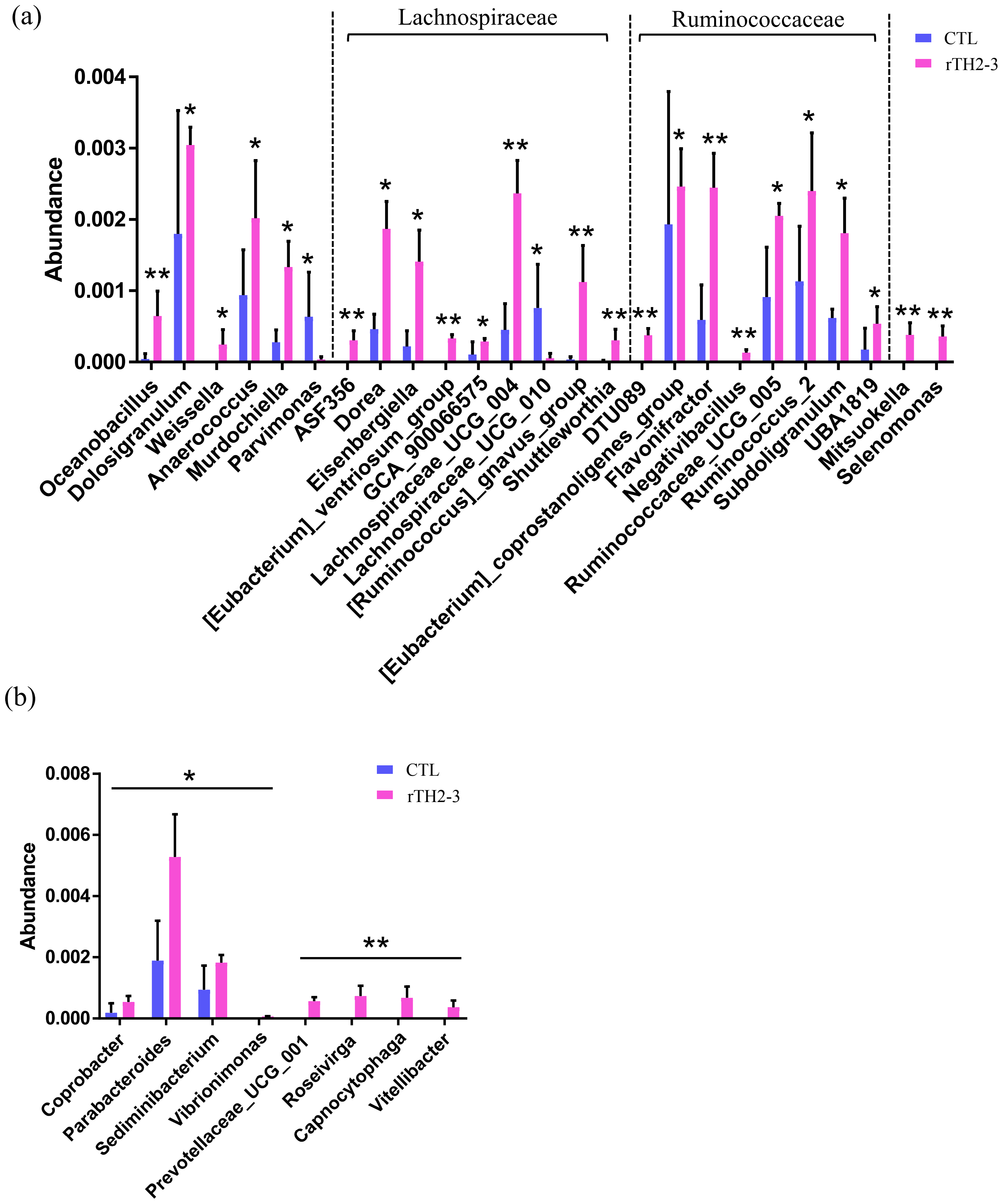

Supplementary Figure 2

(a)

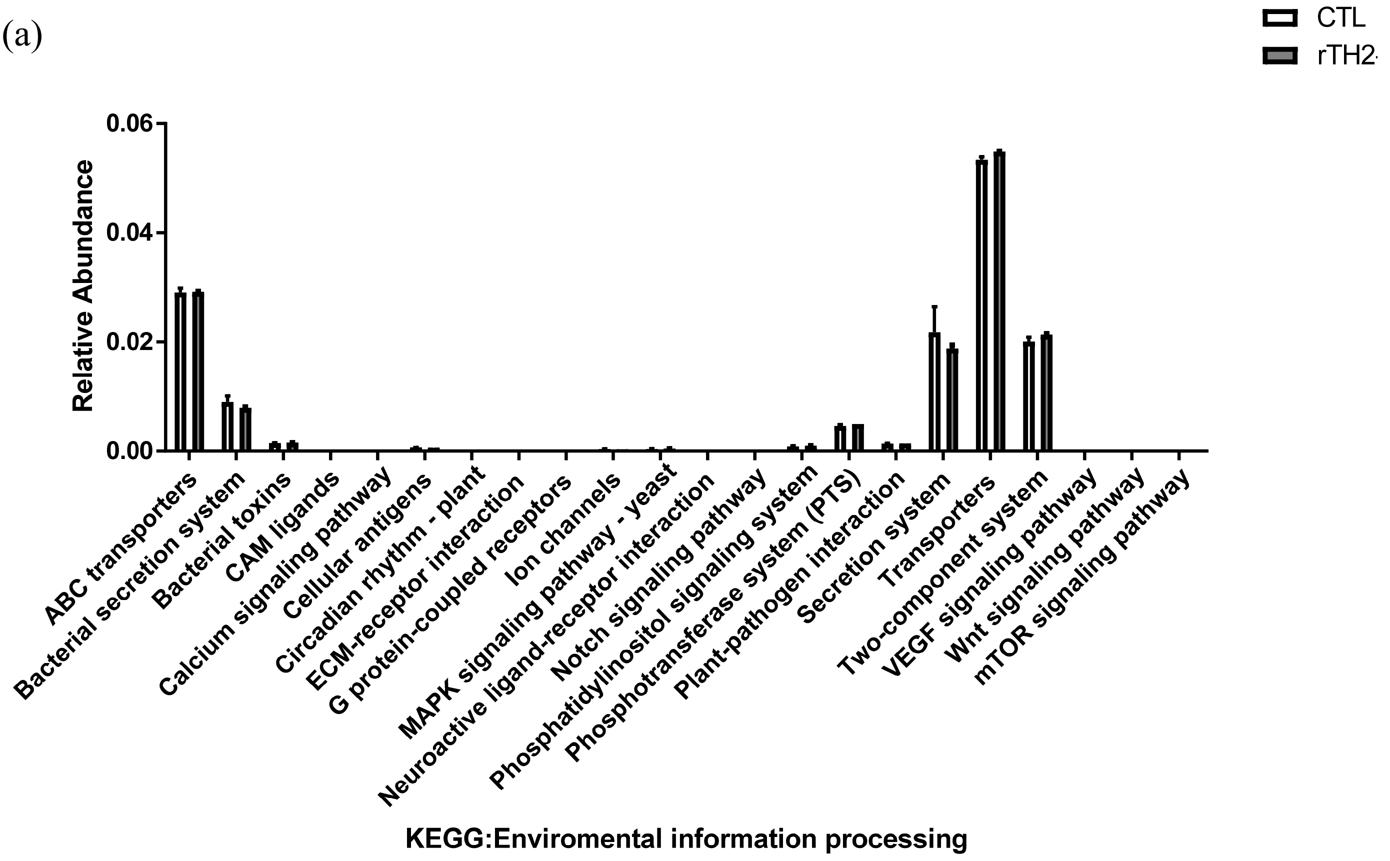

(b)

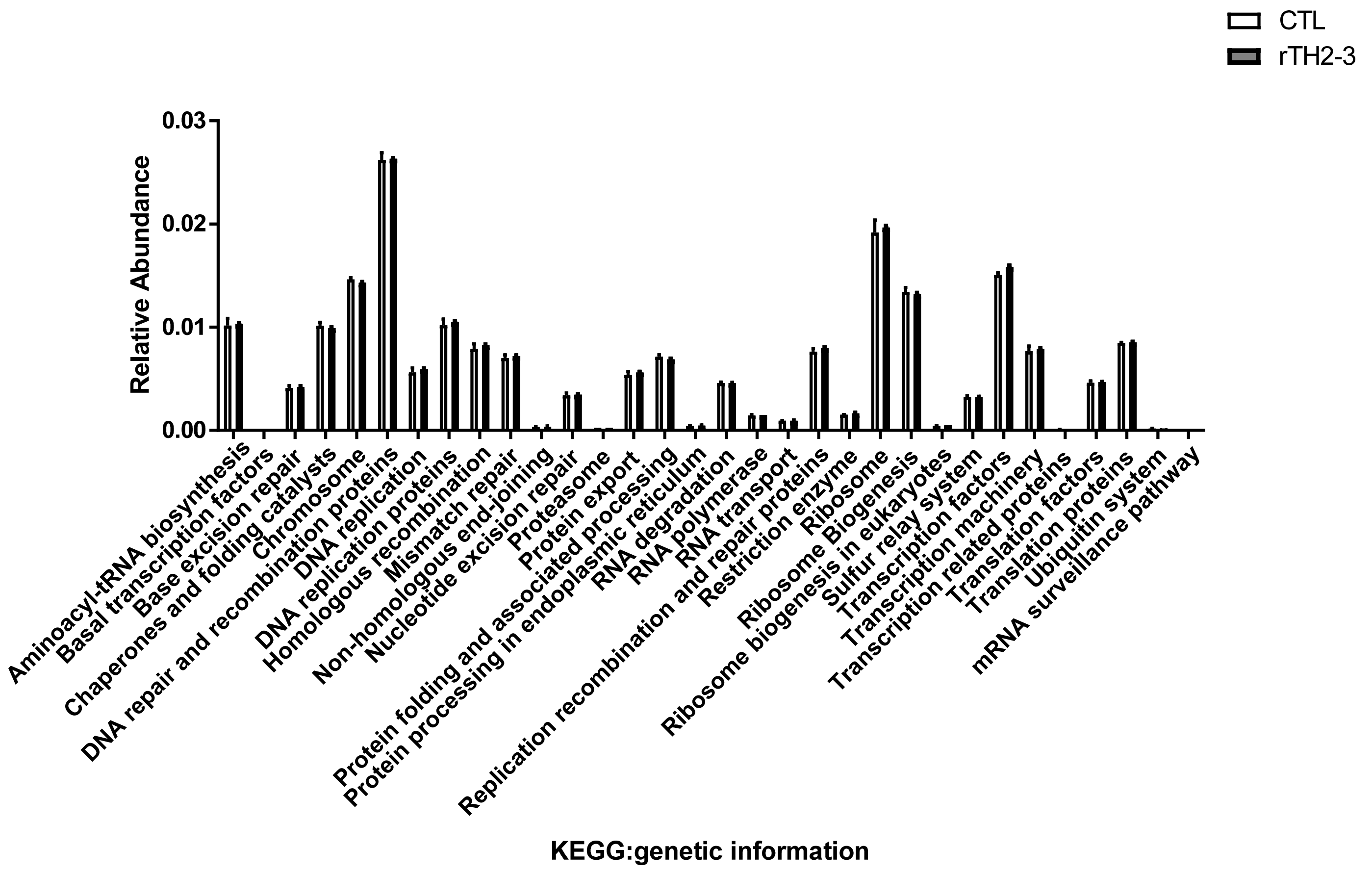

Supplementary Figure 2

(c)

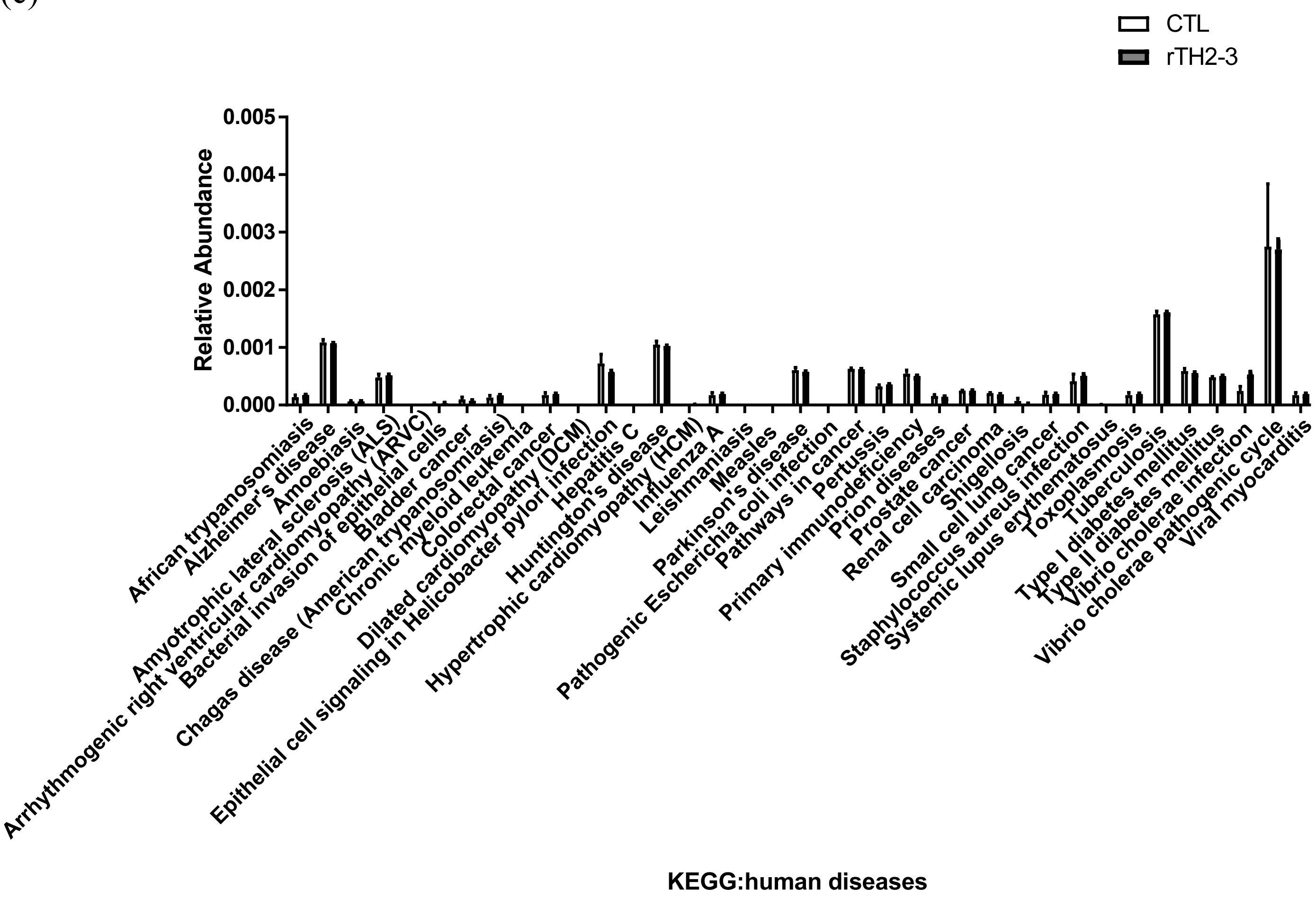

(d)

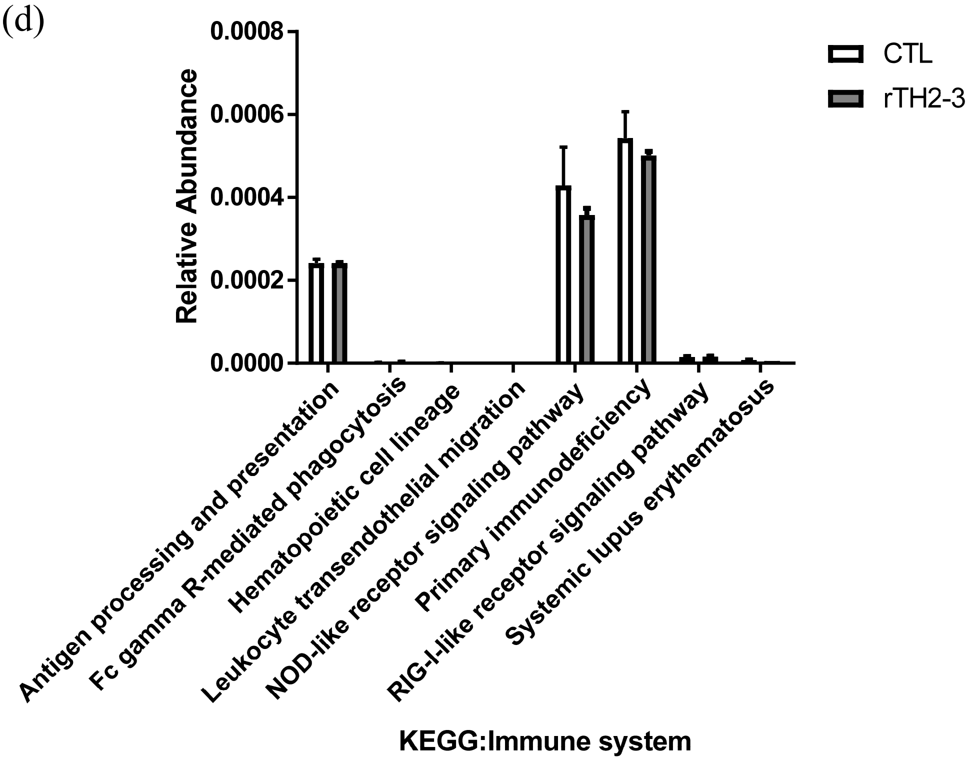

Supplementary Figure 2

(e)

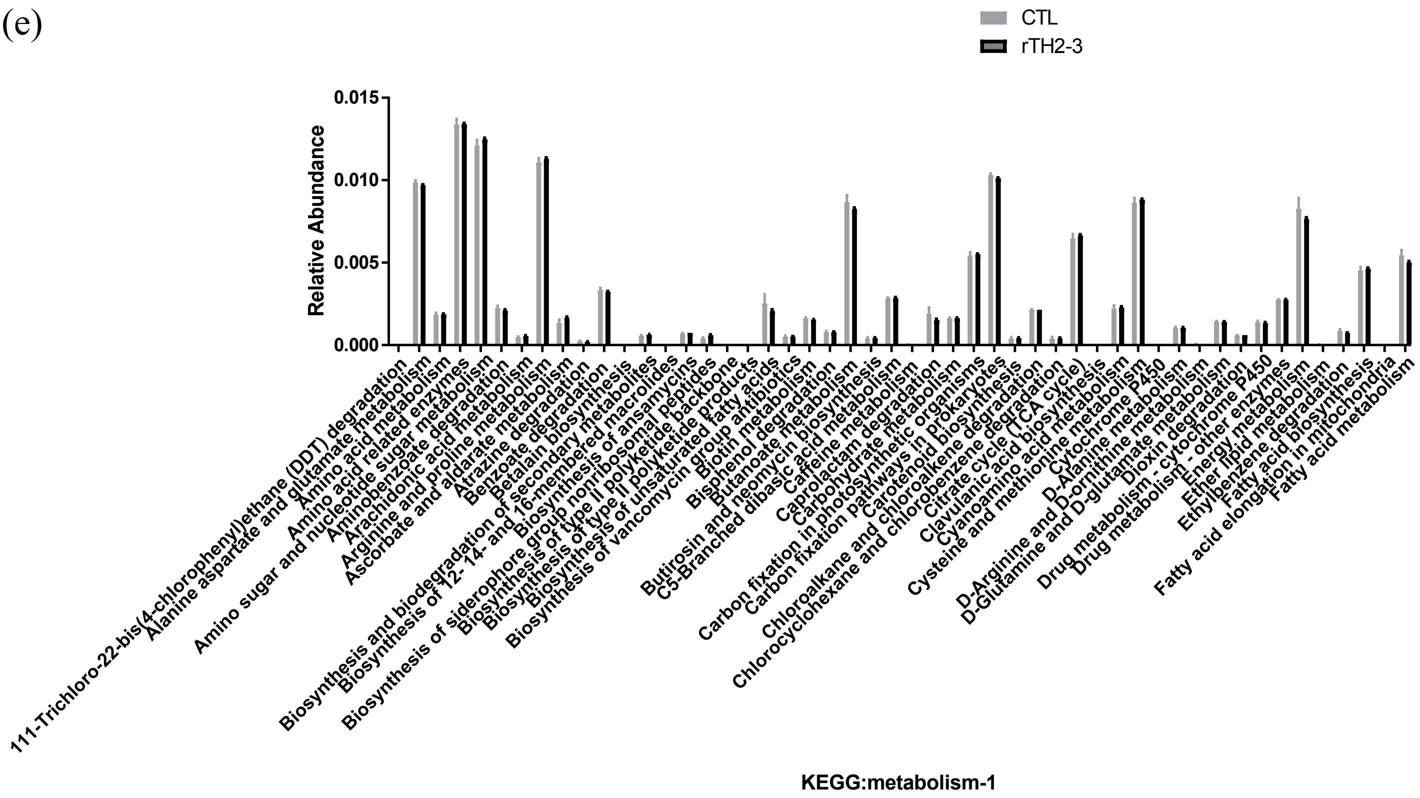

(f)

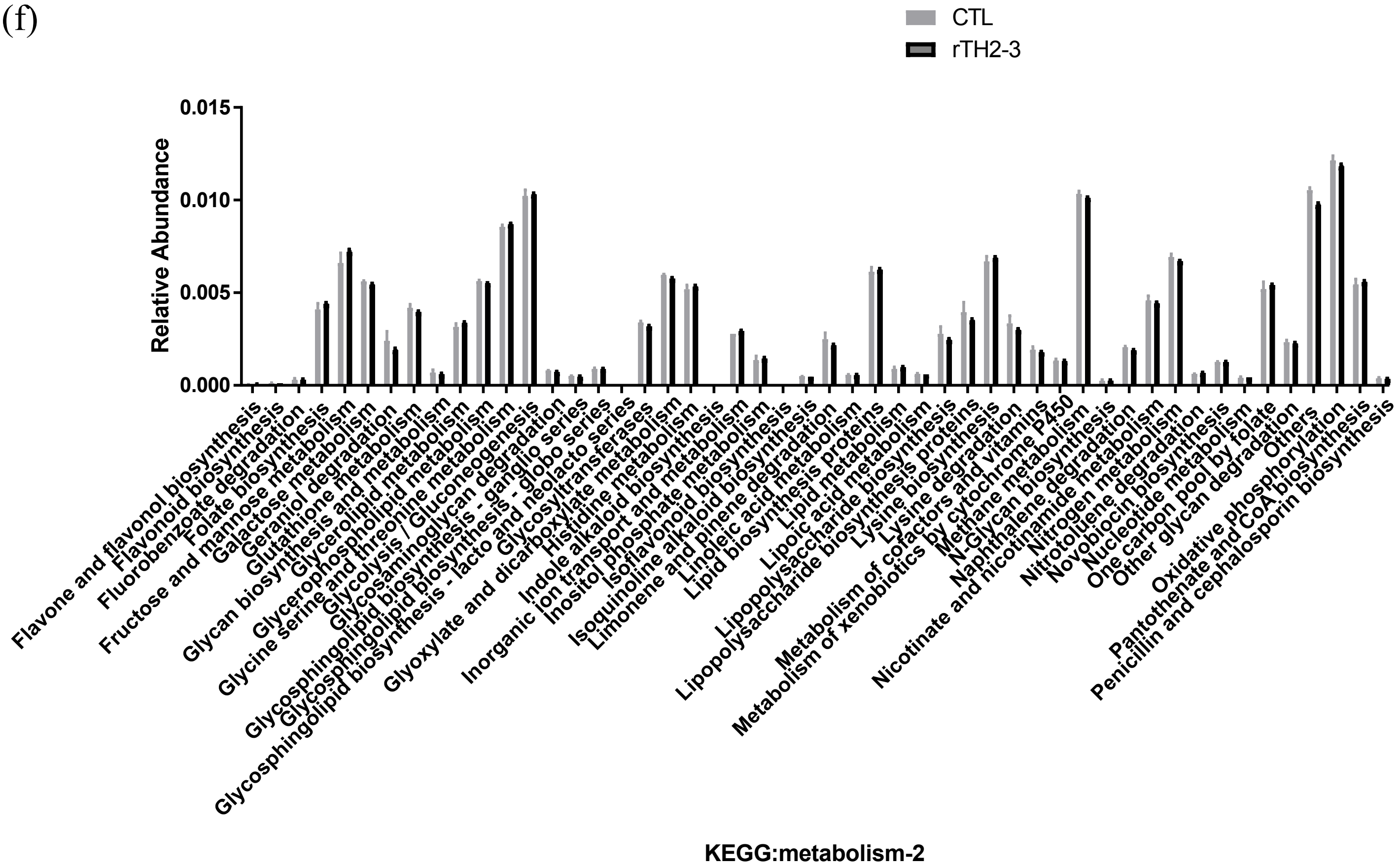

Supplementary Figure 2

(g)

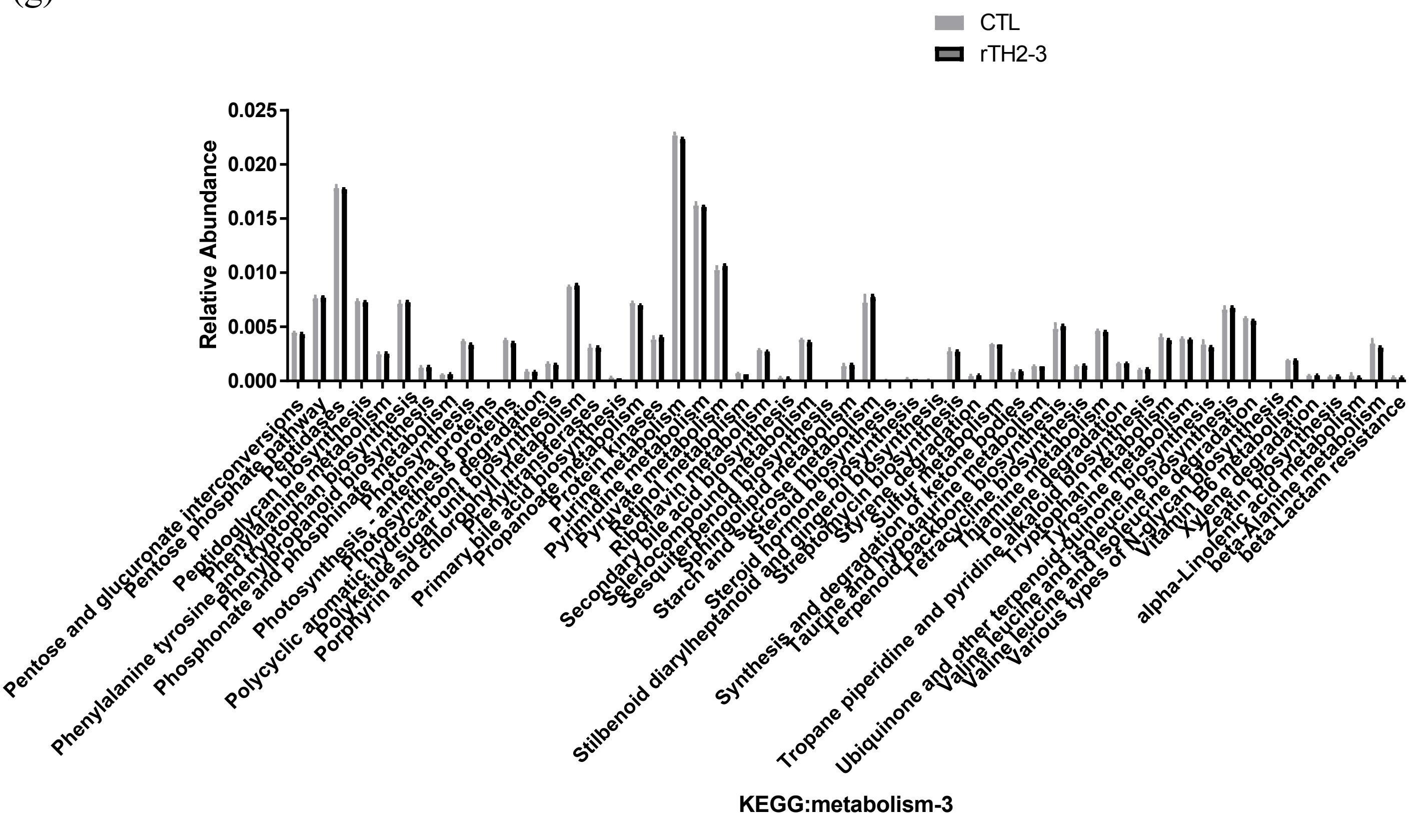

Supplement: Supplementary file 1 — supplementary information [file 41598_2019_55509_MOESM1_ESM.pdf]
